# Enhanced cardiac repair by telomerase reverse transcriptase over-expression in human cardiac mesenchymal stromal cells

**DOI:** 10.1038/s41598-019-47022-w

**Published:** 2019-07-22

**Authors:** Thi Yen Loan Le, Hilda A. Pickett, Andrian Yang, Joshua W. K. Ho, Sujitha Thavapalachandran, Sindhu Igoor, Sile F. Yang, Melad Farraha, Holly K. Voges, James E. Hudson, Cristobal G. dos Remedios, Tracy M. Bryan, Eddy Kizana, James J. H. Chong

**Affiliations:** 1 Centre for Heart Research, Westmead Institute for Medical Research, The University of Sydney, Westmead, NSW 2145 Australia; 2 Department of Cardiology, Westmead Hospital, Westmead, NSW 2145 Australia; 3 Telomere Length Regulation Unit, Children’s Medical Research Institute, The University of Sydney, Westmead, NSW 2145 Australia; 4 Victor Chang Cardiac Research Institute, and St Vincent’s Clinical School, University of New South Wales, Darlinghurst, NSW 2010 Australia; 5 Sydney Medical School, The University of Sydney, Sydney, NSW 2006 Australia; 6 School of Biomedical Sciences, University of Queensland, St Lucia, Queensland 4072 Australia; 7 Department of Anatomy and Histology, School of Medical Sciences, Bosch Institute, The University of Sydney, Sydney, NSW 2006 Australia; 8 Cell Biology Unit, Children’s Medical Research Institute, The University of Sydney, Westmead, NSW 2145 Australia; 9 Victor Chang Cardiac Research Institute, Darlinghurst, NSW 2010 Australia

**Keywords:** Cardiac regeneration, Heart stem cells

## Abstract

We have previously reported a subpopulation of mesenchymal stromal cells (MSCs) within the platelet-derived growth factor receptor-alpha (PDGFRα)/CD90 co-expressing cardiac interstitial and adventitial cell fraction. Here we further characterise PDGFRα/CD90-expressing cardiac MSCs (PDGFRα + cMSCs) and use human telomerase reverse transcriptase (hTERT) over-expression to increase cMSCs ability to repair the heart after induced myocardial infarction. hTERT over-expression in PDGFRα + cardiac MSCs (hTERT + PDGFRα + cMSCs) modulates cell differentiation, proliferation, survival and angiogenesis related genes. *In vivo*, transplantation of hTERT + PDGFRα + cMSCs in athymic rats significantly increased left ventricular function, reduced scar size, increased angiogenesis and proliferation of both cardiomyocyte and non-myocyte cell fractions four weeks after myocardial infarction. In contrast, transplantation of mutant hTERT + PDGFRα + cMSCs (which generate catalytically-inactive telomerase) failed to replicate this cardiac functional improvement, indicating a telomerase-dependent mechanism. There was no hTERT + PDGFRα + cMSCs engraftment 14 days after transplantation indicating functional improvement occurred by paracrine mechanisms. Mass spectrometry on hTERT + PDGFRα + cMSCs conditioned media showed increased proteins associated with matrix modulation, angiogenesis, cell proliferation/survival/adhesion and innate immunity function. Our study shows that hTERT can activate pro-regenerative signalling within PDGFRα + cMSCs and enhance cardiac repair after myocardial infarction. An increased understanding of hTERT’s role in mesenchymal stromal cells from various organs will favourably impact clinical regenerative and anti-cancer therapies.

## Introduction

Despite considerable advances in myocardial infarction (MI) and heart failure (HF) treatment, morbidity and mortality after MI remain an increasing problem^1^. Our growing understanding of cardiac cellular composition^2^ and cell biology^3^ raises intriguing future therapeutic possibilities. Specifically, cardiac fibroblasts, a heterogeneous population potentially including progenitor cells, may be permissive to manipulation for cardiac repair^4^.

Progenitor and stem cell senescence is regulated by telomerase activity and telomere length^5,6^. Telomeres are DNA protein structures that protect chromosome ends from degradation and fusion. Telomerase is a ribonucleoprotein complex that maintains telomere length^7^. Dysfunction of telomerase and telomere shortening are associated with impaired tissue repair in pathological conditions including ageing, HF and MI^8,9^. Furthermore, telomerase activation, by expressing human telomerase reverse transcriptase (hTERT), has been explored as a strategy to elongate telomeres and rejuvenate aged stem cells^10–12^. *Tert* transgenic expression promotes cardiomyocyte proliferation, hypertrophy and survival^13^ and *Tert* gene therapy delays ageing^14^. Therefore TERT manipulation presents a promising method to improve progenitor cell function.

We have previously described mesenchymal stromal cell (MSC)-like cells within adult murine^15^ and human hearts^16^. These cardiac mesenchymal stromal cells (cMSCs) reside within the cardiac interstitium and adventitia of coronary arteries^15,16^. Through genetic lineage tracing, we have shown these cMSCs derive from the embryonic epicardium during heart development and, not from bone marrow (BM)^15^. We have shown that a cell-sorting strategy selecting the platelet-derived growth factor receptor-alpha (PDGFRα+)/CD90+/CD31^−^ fraction enriches for cells that have a MSC phenotype^17^. We hypothesise that these PDGFRα-expressing cMSCs (PDGFRα + cMSCs) are linked to cardiac disease through processes of inflammation and fibrosis, and therefore represent potential therapeutic targets.

In the present study, we characterise PDGFRα + cMSCs derived from human hearts, and demonstrate that over-expression of hTERT increases plasticity of both aged and disease-related phenotypes. hTERT induced telomerase activity increased telomere length. Growth kinetics, cell proliferation, survival and differentiation were enhanced by hTERT over-expression. *In vivo*, transplantation of hTERT + PDGFRα + cMSCs improved cardiac function by decreasing scar size and pro-fibrotic factors, increasing angiogenesis, and enhancing both cardiomyocyte (CM) and non-myocyte cell proliferation. These findings shed light on hTERT’s role in mesenchymal progenitors and are proof-of-principle that cell therapy augmented with hTERT over-expression can be an effective means to promote cardiac repair.

## Results

### Human PDGFRα-expressing cardiac mesenchymal stromal cells (PDGFRα + cMSCs) express both fibroblast and MSC markers

To investigate the effects of age and disease on human PDGFRα + cMSCs^16,17^, we isolated and characterised cells from young (6 ± 3 years), adult (61 ± 3 years) and diseased (56 ± 2 years) hearts as previously described^17^. Microscopy showed no morphological differences between cells from aged or diseased samples which at baseline (no sort), expressed fibroblast and cardiac progenitor markers, (including vimentin, PDGFRα, PDGFRβ, CD90 and c-Kit; Supplementary Fig. S1). No expression of the pan-hematopoietic marker CD45 was found. To enrich for cMSCs, fluorescence-activated cell sorting (FACS) for the PDGFRα+/CD90+/CD31^−^ fraction was performed^17^.

**Figure 1:**
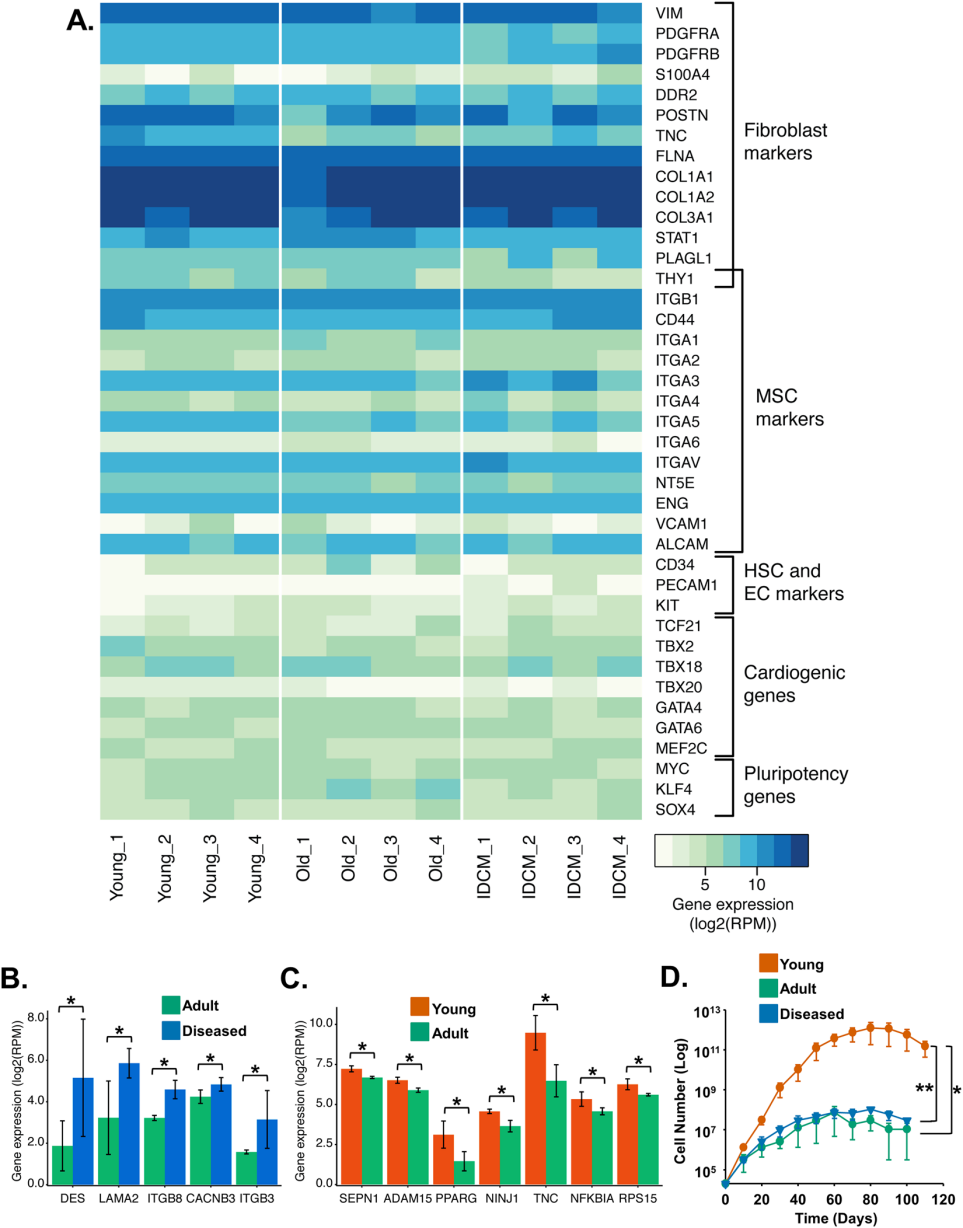
Human PDGFRα + cMSCs derived from young, adult and diseased hearts express defined cardiac fibroblast and MSC markers. **(A)** Heat map of RNAseq analysis showing expression of known fibroblast and MSC markers, as well as cardiogenic and pluripotency genes in PDGFRα + cMSCs derived from young, adult and diseased hearts. High expression of genes shown in blue and low expression in white. **(B)** Gene ontology analysis shows up-regulation of genes associated with dilated cardiomyopathy in diseased compared to non-diseased cells. **(C)** Gene ontology analysis showing up-regulation of regenerative genes in cells derived from young compared to adult hearts. **(D)** Growth-curve analysis showing cell number decrease with age/disease in PDGFRα + cMSCs. N = 4 patient samples/group. Data presented as Mean ± SEM; ns, not significant, *p < 0.05, **p < 0.005, using one-way ANOVA with Holm-Sidak post-test.

We then performed high-throughput next-generation RNA-sequencing (RNAseq) of sorted PDGFRα + cMSCs. We observed high enrichment of fibroblast markers, MSC markers and cardiogenic transcription factors (Fig. 1A). Interestingly, PDGFRα + cMSCs expressed *MYC*, *KLF4* and *SOX4*, which are implicated in self-renewal and pluripotency of stem cells (Fig. 1A). As expected, no significant expression of haematopoietic and endothelial cell markers (CD45, PECAM1, Fig. 1A) was seen. RNAseq was validated by quantitative reverse transcription polymerase chain reactions (qRT-PCR) (Supplementary Fig. S2). Among these transcripts, *CD90* and *TNC* were more highly expressed in young (~3-fold and ~3.5-fold, respectively) compared to adult and diseased cells (Supplementary Fig. S2), suggesting an enrichment for MSCs in young over adult or diseased hearts. Together, these data suggest enrichment of progenitor cells within the PDGFRα + cMSC population.

Functional annotation of RNAseq analysis was performed to identify molecular differences between young and adult non-diseased as well as diseased PDGFRα + cMSCs. Genes associated with dilated cardiomyopathy (*DES, LAMA2, ITGB8, CACNB3* and *ITGB3*)^18,19^ showed higher expression in diseased compared to age-matched, non-diseased hearts (Fig. 1B). This possibly implicates an underappreciated role of mesenchymal cells in the cardiomyopathic disease process. Gene ontology (GO) term analysis identified increased expression of tissue regeneration genes in young compared to adult PDGFRα + cMSCs (Fig. 1C), supporting the use of young cMSCs for further cell transplantation experiments.

Long-term self-renewal of PDGFRα + cMSCs in all groups was quantified by growth-curve analysis of serial passaging (Fig. 1D). PDGFRα + cMSCs grew for at least 3 months (approximately 10 successive passages) but this was significantly reduced in adult and diseased PDGFRα + cMSCs (Fig. 1D). We have previously reported that this decreased proliferative activity of aged PDGFRα + cMSCs correlates with shorter telomere lengths in the absence of telomerase activity^17^. Therefore, we hypothesised that manipulation of hTERT in PDGFRα + cMSCs might restore their proliferative potential and confer a regenerative phenotype as suggested by our RNAseq data.

### hTERT over-expression results in telomerase activity and increases telomere length in PDGFRα + cMSCs

hTERT, the rate-limiting catalytic enzyme of the nucleoprotein complex telomerase, has a well-defined role in telomere maintenance, cell proliferation and renewal. However, increasing reports document important non-canonical (unrelated to telomere length maintenance) functions of hTERT in progenitor cell populations^20,21^. Therefore, we used qRT-PCR and telomeric repeat amplification protocol (TRAP) assays to investigate the presence of hTERT/telomerase in PDGFRα + cMSCs.

**Figure 2:**
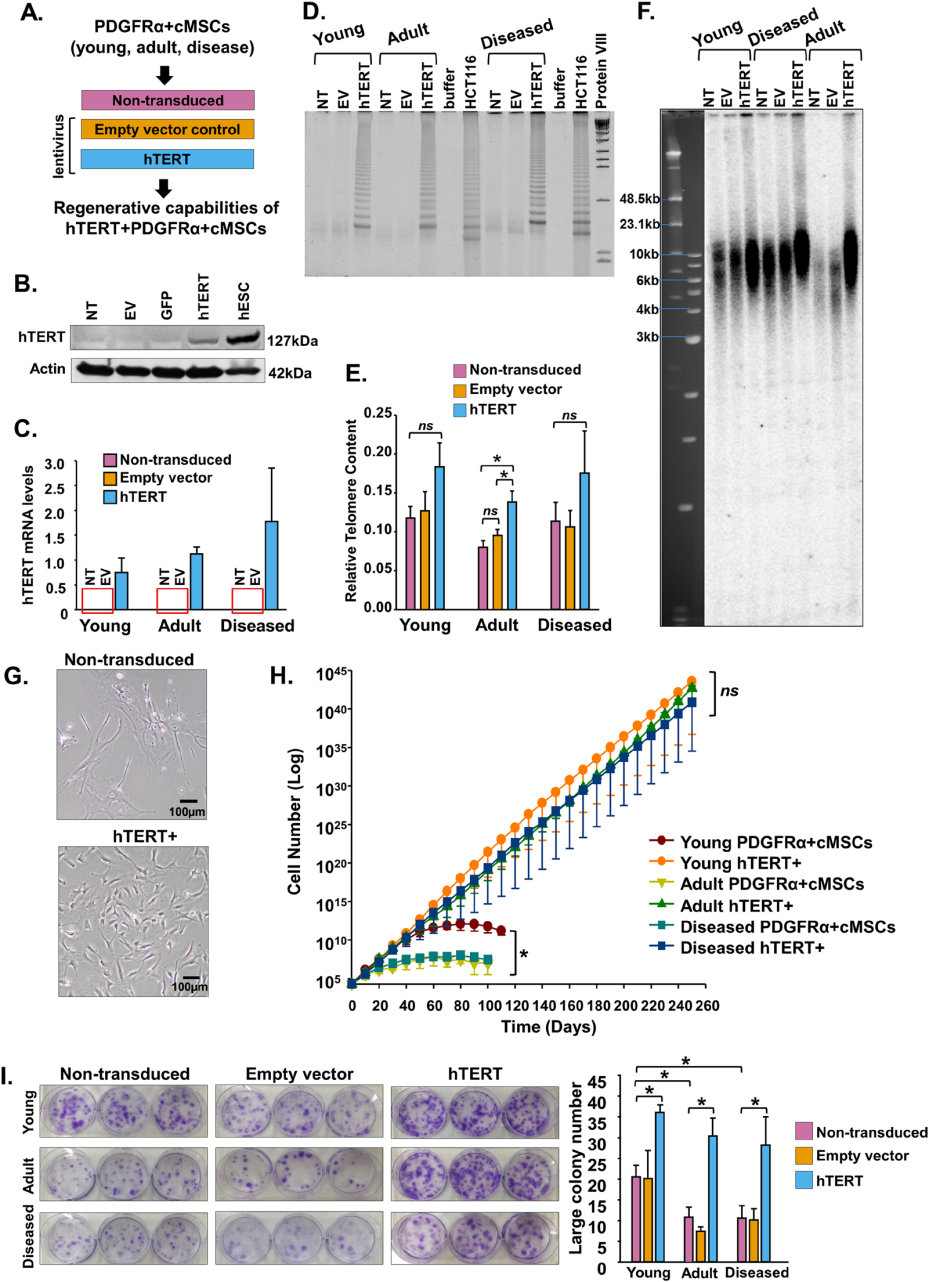
Expression of hTERT in PDGFRα + cMSCs rescues age-related decline of PDGFRα + cMSC growth potential and colony formation. (**A)** PDGFRα + cMSCs from young, adult and diseased hearts were transduced with lentivirus containing hTERT gene or controls (non-transduced [NT], empty vector [EV] and Green Fluorescent Protein [GFP]). **(B,C)** Expression of hTERT in PDGFRα + cMSCs confirmed by qRT-PCR and Western blot assays. hESC, human embryonic stem cells. **(D)** Telomeric repeat amplification protocol (TRAP) assay showing no detectable telomerase in non-transduced cells from diseased, young and adult hearts but induced telomerase activity after hTERT over-expression. HCT116 cell line and buffer only were used as telomerase positive and negative controls, respectively. **(E,F)** Analysis of telomere length by telomere-qPCR and terminal restriction fragment (TRF) length analysis. **(G)** hTERT over-expression induces a spindle-shaped morphological change in PDGFRα + cMSCs. **(H)** Extended long-term growth of PDGFRα + cMSCs from young, adult and diseased hearts transduced with hTERT. **(I)** Colony-forming unit-fibroblast assays show decreased colony forming ability in PDGFRα + cMSCs from adult and diseased hearts. This is rejuvenated after hTERT over-expression as seen by increased large colony numbers. N = 4 patient samples/group. Data presented as Mean ± SEM; ns, not significant, *p < 0.05, using one-way ANOVA with Holm-Sidak post-test. See also Supplementary Figs S3, S4.

In agreement with other reports^22^, PDGFRα + cMSCs from young, adult and diseased hearts did not express hTERT or telomerase (data not shown). In the absence of telomerase activity, telomeres progressively shorten with each cell division until a state of cellular senescence is reached. In order to delay this senescence, we used a lentiviral system to stably over-express hTERT in PDGFRα + cMSCs from young, adult and diseased hearts. Empty vector (EV)-transduced PDGFRα + cMSCs were used as controls (Fig. 2A). We confirmed over-expression of exogenous hTERT in PDGFRα + cMSCs by Western blot (Fig. 2B) and qRT-PCR (Fig. 2C), with undetectable hTERT in non-transduced (NT) cells, EV and GFP controls.

Telomerase comprises hTERT reverse transcriptase and hTR RNA (template-driven telomere synthesis region). hTR is ubiquitously expressed, and we confirmed that expression of hTR was not significantly altered following hTERT over-expression (Supplementary Fig. S3A). Both hTR and hTERT components are required for telomerase activity. Next we used the TRAP assay to confirm over-expression of hTERT induced telomerase activity (Figs 2D, S3B).

Because hTERT-induced telomerase activity is required for telomere maintenance and unlimited cellular division, we examined telomere lengths in PDGFRα + cMSCs by telomere-qPCR (Fig. 2E) and by terminal restriction fragment (TRF) length analysis (Fig. 2F). As expected, we observed increased telomere length in hTERT + PDGFRα + cMSCs compared with NT and EV controls (Fig. 2E,F). This demonstrates that hTERT expression in PDGFRα + cMSCs results in telomerase activity and telomere elongation. Importantly, hTERT expression did not alter the normal karyotype of PDGFRα + cMSCs (Supplementary Fig. S3C). These data confirm canonical functions of telomerase following hTERT over-expression in PDGFRα + cMSCs.

### hTERT over-expression rescues age-related decline of PDGFRα + cMSC self-renewal, colony formation, proliferation and survival

The presence of hTERT and telomerase is important for self-renewal of cells. Consistent with a differentiated phenotype, PDGFRα + cMSCs eventually became morphologically broad and ceased to proliferate; in contrast, hTERT + PDGFRα + cMSCs maintained spindle-shaped morphology and continued long-term self-renewal without senescence (Fig. 2G,H). To further examine the impact of hTERT on PDGFRα + cMSC self-renewal, we performed colony-forming assays where clonogenicity and proliferation of cells derived from large (>2 mm) colonies exhibit the greatest multipotency and greatest long-term growth capacity^15^. Consistent with our previous studies, PDGFRα + cMSCs formed colonies with a range of sizes (Supplementary Fig. S4A). PDGFRα + cMSCs from the aged and diseased samples led to fewer large colonies compared to young hearts (Fig. 2I). In contrast, hTERT + PDGFRα + cMSCs possessed significantly higher large-colony formation independent of the age or disease status of hearts from which they were isolated (Fig. 2I). These results, together with the growth-curve analysis (Fig. 2H), suggest hTERT over-expression in PDGFRα + cMSCs rejuvenates progenitor cell properties and replicates a young heart phenotype.

**Figure 3:**
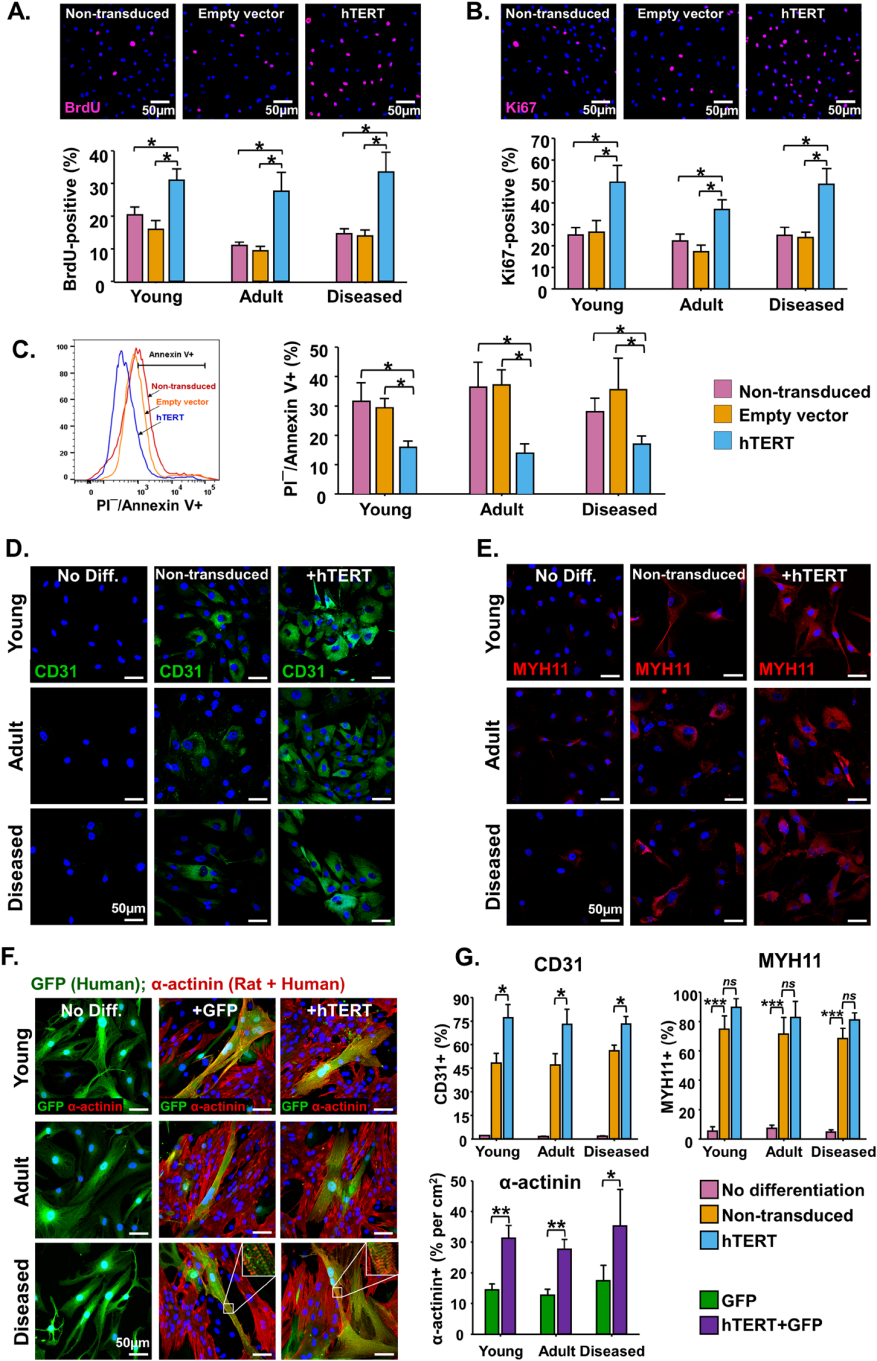
hTERT over-expression enhances cell proliferation, survival and increases vascular and myocyte protein expression. (**A,B)** Representative images of BrdU and Ki67 stained cells. Quantification in bar graphs of BrdU and Ki67-positive cells show increased cell cycle activity in hTERT + PDGFRα + cMSCs. **(C)** Representative flow cytometry analysis of Propidium Iodide/Annexin V and quantification of starvation-induced apoptotic cell death in hTERT-transduced PDGFRα + cMSCs compared to non-transduced PDGFRα + cMSCs and empty vector controls. **(D,E)** Representative images of PDGFRα + cMSCs subjected to endothelial and smooth muscle cell differentiation assays then stained with CD31 and smooth muscle myosin heavy chain 11 (MYH11). **(F)** Representative images of GFP-transduced PDGFRα + cMSCs co-cultured with neonatal rat ventricular myocytes (NRVMs) for 14 days then stained for α-actinin. Green = GFP (Human cells); Red = α-actinin (Human cells and NRVMs). **(G)** Bar graphs show quantification of the CD31, MYH11 and α-actinin positive cells in differentiation assays. N = 4 patient samples/group. Data presented as Mean ± SEM; ns, not significant, *p < 0.05, **p < 0.01, ***p < 0.001, using unpaired Student’s t-test or one-way ANOVA for multiple comparisons. See also Supplementary Fig. S5.

Next, we examined the effects of hTERT over-expression on cell proliferation. The proliferative activity of PDGFRα + cMSCs was assessed by incorporation of BrdU (Fig. 3A) and Ki67 immunostaining (Fig. 3B). Levels of BrdU incorporation (Fig. 3A) and Ki67 (Fig. 3B) were significantly (p < 0.05) higher in hTERT + PDGFRα + cMSCs compared to NT and EV controls. These results support our growth-curve (Fig. 2H) and colony formation (Fig. 2I) data, showing hTERT over-expression induces a highly proliferative phenotype in PDGFRα + cMSCs.

We next examined the effects of hTERT over-expression on apoptosis of PDGFRα + cMSCs. In response to starvation-induced apoptosis, the propidium iodide (PI)-negative/annexin V-positive cell fraction (early apoptosis) was significantly decreased in hTERT + PDGFRα + cMSCs (Fig. 3C), consistent with previous studies^10^. Together, these data show that hTERT over-expression can increase proliferation and survival of PDGFRα + cMSCs. This strategy may be useful for cardiac cell therapy approaches.

### Over-expression of hTERT in PDGFRα + cMSCs increases endothelial cell, smooth muscle cell and myocyte protein expression

To examine whether over-expression of hTERT influences PDGFRα + cMSC differentiation, we performed *in vitro* vascular (endothelial and smooth muscle) and myocyte differentiation assays on non-hTERT and hTERT-transduced cells. After 14 days of endothelial cell differentiation, there were significantly higher levels of CD31 protein expression in the hTERT + PDGFRα + cMSC compared to PDGFRα + cMSC groups (Fig. 3D,G). In contrast to endothelial cell differentiation, hTERT over-expression only slightly increased PDGF-BB-induced smooth muscle cell protein expression (MYH11 + ) (Fig. 3E,G). These data suggest that hTERT over-expression enhances PDGFRα + cMSC endothelial cell differentiation, which can be exploited for angiogenesis in therapeutic strategies.

Next, we examined the effects of hTERT over-expression on cardiomyocyte differentiation. There was no expression of either sarcomeric α-actinin (Fig. 3F) or cardiac troponin T (cTnT) (Supplementary Fig. S5A) when GFP-transduced PDGFRα + cMSCs were cultured in basal medium alone (without neonatal rat ventricular myocytes [NRVMs]). In contrast, 14 days after co-culture with NRVMs, we observed an increase in α-actinin (Fig. 3F) and cTnT (Supplementary Fig. S5A) protein expression in GFP + PDGFRα + cMSCs. The levels of α-actinin + and cTnT + was significantly higher in hTERT + GFP + PDGFRα + cMSCs compared with GFP + PDGFRα + cMSCs controls (Figs 3G, S5A). There was no cell fusion in our co-culture system, as shown by human nuclei co-immunostaining with only cTnT and α-actinin (Supplementary Fig. S5B). Together these results demonstrate that hTERT over-expression can enhance the vascular and cardiomyocyte protein expression in PDGFRα + cMSCs.

### hTERT changes PDGFRα + cMSC transcriptional profiles towards a stem cell/progenitor phenotype

To examine how hTERT over-expression induces cellular changes in the experiments above, we performed RNAseq on hTERT-over-expressing PDGFRα + cMSCs from young, adult and diseased human hearts. NT and EV-transduced PDGFRα + cMSCs were again used as controls. The gene expression profiles of 11,802 genes were examined after removal of duplicated genes following transcript alignment. Genes in hTERT+ samples were considered as significantly differentially expressed if they had an absolute fold change >1 and p < 0.05 compared to the NT samples in addition to the same genes not being significantly differentially expressed in the EV-NT controls. A total of 721 (young), 433 (adult) and 414 (diseased) genes were differentially expressed in hTERT + PDGFRα + cMSCs versus controls (NT and EV). Of these, 230 (young), 93 (adult) and 156 (diseased) genes were up-regulated and 491 (young), 340 (adult) and 258 (diseased) were down-regulated in hTERT + PDGFRα + cMSCs, compared to their respective controls. Interestingly, the higher number of up- and down-regulated transcripts in the young (compared to adult and diseased PDGFRα + cMSCs) suggests a more plastic phenotype more permissive to hTERT-induced change.

**Figure 4:**
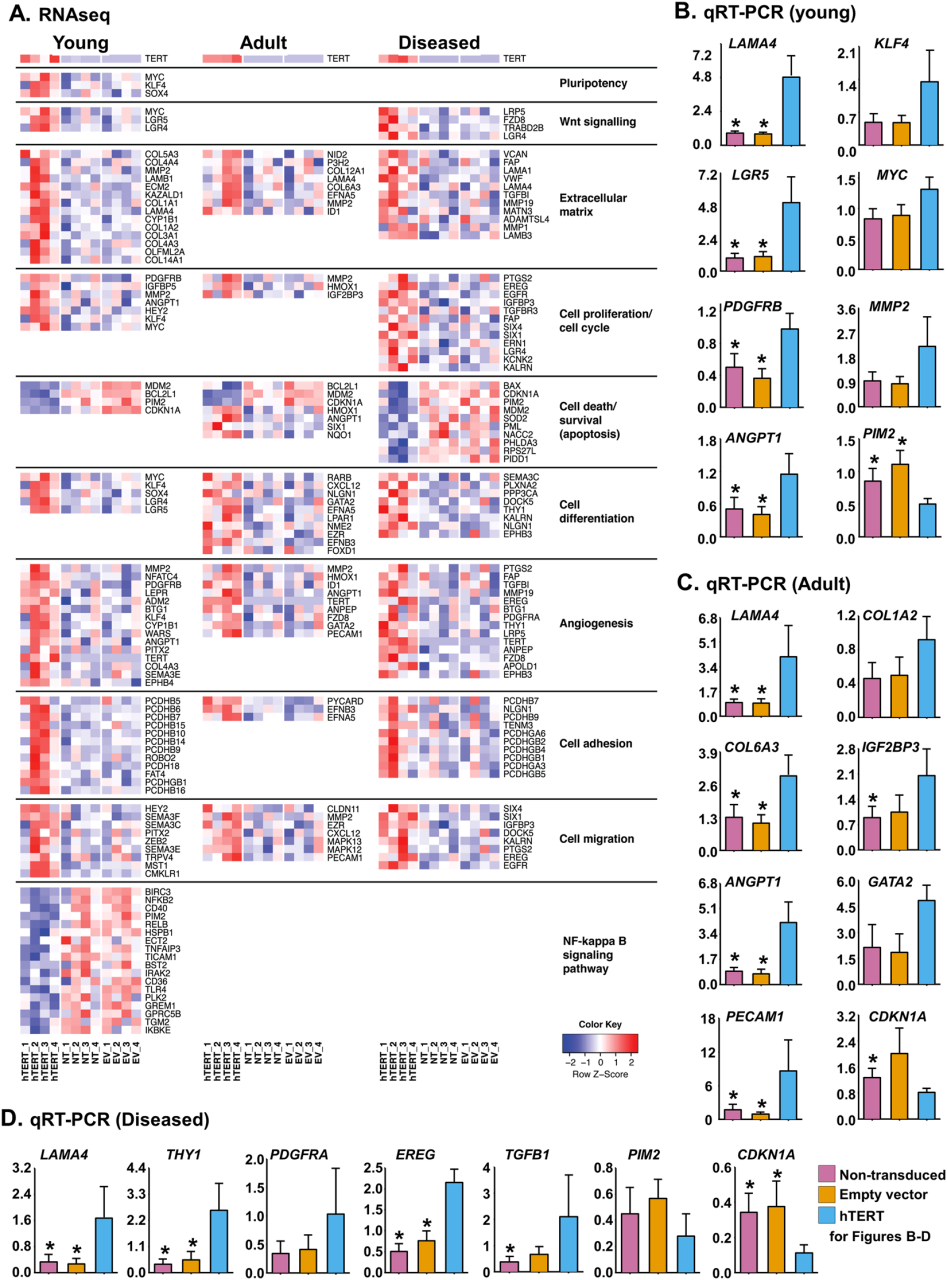
Transcriptome profile of hTERT-expressing PDGFRα + cMSCs. (**A)** Gene expression changes in hTERT + PDGFRα + cMSCs, non-transduced (NT) and empty vector (EV) controls from young, adult and diseased hearts determined by next-generation RNA-sequencing (RNAseq). Heat map shows up-regulated genes in red and down-regulated genes in blue. Arrangement by gene ontology terms (right). **(B–D)** qRT-PCR confirmation of selected genes from RNAseq. N = 4 patient samples/group. Data presented as Mean ± SEM; *p < 0.05 versus hTERT, using one-way ANOVA with Holm-Sidak post-test. See also Supplementary Fig. S6.

To better characterise the molecular basis of differences between non-hTERT and hTERT + PDGFRα + cMSCs, we used Gene Ontology (GO) enrichment analysis. This functionally annotates and predicts the biological roles of differentially expressed genes (Supplementary Fig. S6). The heat map in Fig. 4A shows change in major genes differentially up-regulated (red) and down-regulated (blue) in non-hTERT versus hTERT + PDGFRα + cMSCs. Biological processes preferentially represented in the hTERT over-expressing cells were associated with Wnt signalling, extracellular matrix (ECM), cell proliferation, survival, differentiation, adhesion, migration and angiogenesis. We validated these findings by qRT-PCR (Fig. 4B,C,D). Although hTERT + PDGFRα + cMSCs from young, adult and diseased hearts display distinct transcriptional profiles, they were all enriched in expression of genes related to cell proliferation, survival and differentiation. This supports our *in vitro* characterisation of growth kinetics, cell cycle activity, apoptosis and differentiation above (Figs 2, 3).

Interestingly, up-regulated genes pointed to networks and pathways of ‘stemness’/pluripotency (*MYC*, *KLF4* and *SOX4*), and down-regulated genes related to NF-ƙB signalling involved in cell stress responses (Fig. 4A). Again, these gene changes were more highly represented in PDGFRα + cMSCs from young hearts and these represent a better cardiac cell therapy candidate.

### hTERT expression in human PDGFRα + cMSCs improves cardiac function and reduces scarring after MI

**Figure 5:**
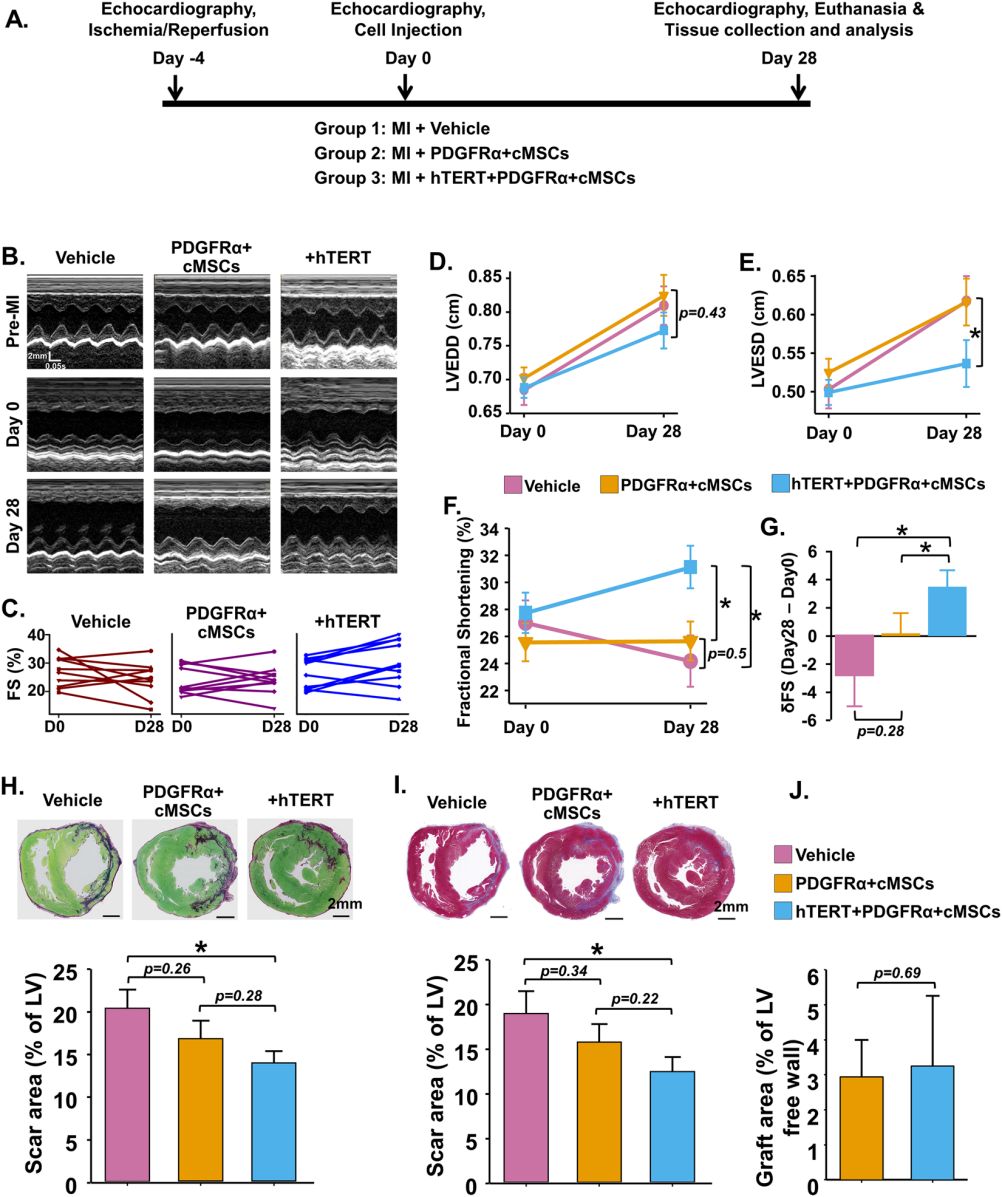
Cardiac functional assessment and histological analysis of PDGFRα + cMSC treated hearts after induced myocardial infarction (MI). (**A**) Experimental design. (**B–G**) Representative M-mode images and consecutive echocardiographic data before and four weeks after cell delivery to rats with myocardial infarction. (**H**) Fibrotic scar area assessed by picroSirius red (purple = fibrotic infarct area; green = non-infarcted area) and (**I**) Gomori Trichrome staining (blue = fibrotic infarct area; red = non-infarcted area). (**J**) Analysis of cell engraftment four days after MI. LVEDD, left ventricular end-diastolic diameter; LVESD, left ventricular end-systolic diameter; FS, fractional shortening; LV, left ventricle. N = 10 rats/group. Data presented as Mean ± SEM, *p < 0.05, using one-way ANOVA with Holm-Sidak post-test. See also Supplementary Fig. S9.

Prior to commencing cell transplantation functional studies, pilot study was first performed to assess the optimal cell-dose. We injected 5 × 10^5^, 5 × 10^6^ and 1 × 10^7^ cells into athymic rat hearts and assessed their impact on animal survival after 2 days. The 5 × 10^5^ dose showed low engraftment, whereas 1 × 10^7^ dose induced animal death after injection (data not shown). Therefore, the 5 × 10^6^ dose was chosen for further experiments. We then assessed whether hTERT + PDGFRα + cMSCs impact heart repair after ischemia-reperfusion injury. Cardiac function was assessed by echocardiography immediately before cell (5 × 10^6^) injection (4 days after MI) and again 4 weeks after cell transplantation (Fig. 5A). All surgery and analysis was performed in a double-blinded manner. Animal groups were randomly allocated. Treatment groups included PDGFRα + cMSCs or hTERT + PDGFRα + cMSCs. Vehicle only was used for controls. As expected, LV end-systolic dimensions were significantly increased in the control group after 1-month (Fig. 5B,D,E), with a corresponding decrease in LV fractional shortening (Fig. 5C,F,G). In contrast, fractional shortening was significantly improved in rats that received hTERT + PDGFRα + cMSCs (p < 0.05) but not in those that received PDGFRα + cMSCs (Fig. 5C,F,G). This supports our *in vitro* and transcriptional profiling experiments, suggesting hTERT + PDGFRα + cMSCs have favourable cardiac repair potential.

To investigate how transplanted hTERT + PDGFRα + cMSCs improve cardiac function, we performed detailed histological analysis, including determination of scar size using PicroSirius Red (Fig. 5H) and Gomori Trichrome staining (Fig. 5I). hTERT + PDGFRα + cMSC-treated rats had smaller infarcts compared with vehicle-only-treated controls (Fig. 5H,I). PDGFRα + cMSCs only slightly decreased scar size (not significant, Fig. 5H,I). There are no significant differences in intraventricular septal and posterior wall thickness in PDGFRα + cMSCs and hTERT + PDGFRα + cMSCs-treated animals compared to vehicle controls (Supplementary Fig. S7). Since transplantation of PDGFRα + cMSCs *in vivo* could result in unanticipated homing to other organs and possible tumour formation, we performed a full necropsy on each animal examining the heart, lung, spleen, kidney and liver. Analysis of hematoxylin and eosin-stain images showed that hTERT + PDGFRα + cMSC treatment did not cause structural change or tumour formation (Supplementary Fig. S8).

### Cardioprotective effects of hTERT + PDGFRα + cMSCs are independent of cell retention and transdifferentiation after MI

To examine the cardiac engraftment of transplanted PDGFRα + cMSCs, we used immunostaining against human nuclei to identify human PDGFRα + cMSCs on cross-sections of cell-treated infarcted rat hearts. We found no engrafted cells 1-month after transplantation in either PDGFRα + cMSCs or hTERT + PDGFRα + cMSCs groups (data not shown). To further detail cell retention, we injected both PDGFRα + cMSCs and hTERT + PDGFRα + cMSCs into non-injured (no MI) athymic rat hearts (Supplementary Fig. S9A). Histological analysis revealed short-term engraftment of both PDGFRα + cMSCs and hTERT + PDGFRα + cMSCs at 2, 7 and 14 days after transplantation (Supplementary Fig. S9A). To further examine whether over-expression of hTERT influenced cell retention after MI, we injected PDGFRα + cMSCs into infarcted hearts and examined cell engraftment 4 days after transplantation (4 days was chosen based on the time-course study above, Supplementary Fig. S9B). We observed no difference in graft area in PDGFRα + cMSCs-injected rats compared to hTERT + PDGFRα + cMSCs-injected rats after 4 days (Figs 5J, S9B).

Although there was short-term (up to 14 days) engraftment of transplanted PDGFRα + cMSCs (Supplementary Fig. S9), co-immunostaining of human nuclei with α-actinin (cardiomyocyte) and CD31 (endothelial cell) showed no differentiation of PDGFRα + cMSCs *in vivo* (Supplementary Fig. S10). Together, these data suggest that cardiac reparative effects of hTERT + PDGFRα + cMSCs are not due to direct long-term engraftment of transplanted cells but are likely to be due to a non-cell autonomous impact on the surrounding infarcted heart environment.

### hTERT + PDGFRα + cMSCs treatment attenuates myofibroblast activation, and increases cellular proliferation, cardiomyocyte contraction and neovascularisation

**Figure 6:**
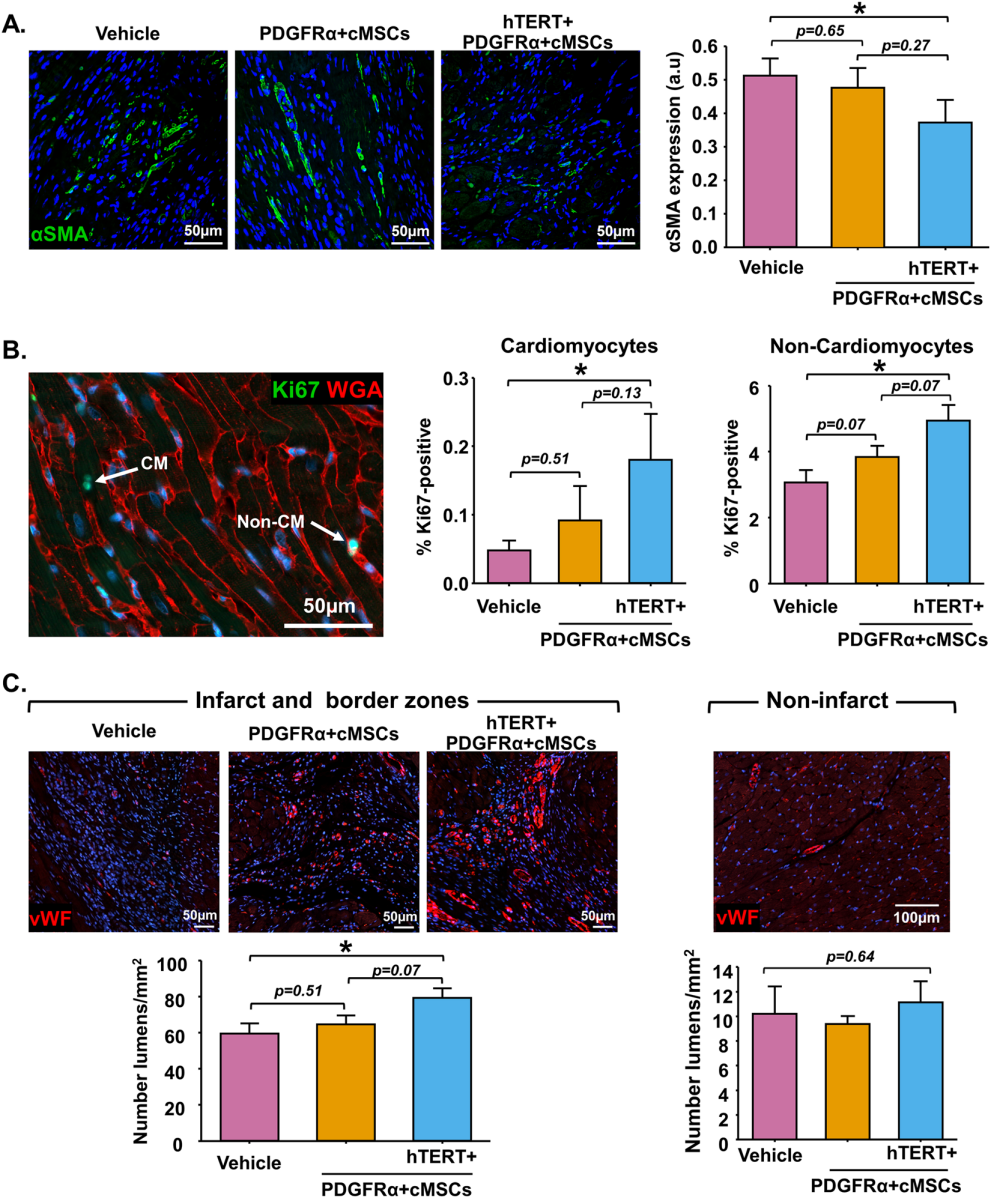
hTERT + PDGFRα + cMSC transplantation decreases myofibroblast activation, increases cardiomyocyte (CM) and non-myocyte proliferation, and enhances angiogenesis. Four weeks post-cell delivery, heart sections were immunostained for (**A**) Alpha-smooth muscle actin (αSMA), (**B**) Ki67 and wheat germ agglutinin (WGA) to mark cardiomyocyte border, (**C**) von Willebrand factor (vWF). Ten random images in different heart regions were selected for quantification in bar graphs. N = 6–10 rats/group. Data presented as Mean ± SEM, *p < 0.05, using one-way ANOVA with Holm-Sidak post-test.

Fibroblast proliferation and differentiation into activated myofibroblasts after MI contributes to increased fibrosis and scar formation^23,24^. We hypothesised that hTERT + PDGFRα + cMSC treatment may attenuate fibroblast differentiation in the infarcted rat myocardium, thus reducing fibrogenic markers such as alpha smooth muscle actin (αSMA). Immunostaining of αSMA showed significantly decreased αSMA protein in the hTERT + PDGFRα + cMSC group (Fig. 6A), but not in the PDGFRα + cMSC group. This suggests that decreased fibrotic scar formation in hTERT + PDGFRα + cMSCs-treated rats (Fig. 5H,I) is due to decreased myofibroblast activation.

To study the impact of hTERT + PDGFRα + cMSC treatment on cardiac cell proliferation after MI, we used Ki67 and WGA co-immunostaining to quantify the percentage of rat cardiomyocytes (CM) and non-cardiomyocytes actively proliferating 4 weeks after hTERT + PDGFRα + cMSC delivery. Minimal Ki67-positive cardiomyocytes and few non-cardiomyocytes were seen in the border area and remote area of the vehicle-treated control group (Fig. 6B). These rates were similar to previous studies^25^. Interestingly, proliferating cardiomyocytes and non-cardiomyocytes were significantly increased in rats that received hTERT + PDGFRα + cMSCs (p < 0.05) but not in those that received PDGFRα + cMSCs (Fig. 6B). Of note, there is no significant difference in the Ki67-positive cardiomyocytes between border and remote areas in all treatment groups (Supplementary Fig. S11).

To further examine how hTERT + PDGFRα + cMSCs interact with and support cardiomyocytes, we co-cultured hTERT + PDGFRα + cMSCs with human cardiomyocytes in a previously validated 3-dimensional (3D) cardiac organoid model (hCO) and measured active force^26,27^. Both PDGFRα + cMSCs and hTERT + PDGFRα + cMSCs integrate throughout the hCO and enhance active force generation by 7 days (Supplementary Fig. S12). However, in contrast to the harsh MI environment, both NT and hTERT + PDGFRα + cMSCs survive very well in the hCO. NT and hTERT + PDGFRα + cMSCs start to overgrow the tissue by 14 days and negatively impact force generation (Supplementary Fig. S12). This illustrates that PDGFRα + cMSCs can directly effect/act on cardiac cells to promote enhanced function, even at the low percentages delivered *in vivo*.

Neovascularisation is essential for maintaining perfusion to damaged myocardium. Therefore we examined blood vessel formation in infarcted hearts after PDGFRα + cMSCs transplantation using von Willebrand factor (vWF) immunostaining to identify blood vessels (Fig. 6C). After infarction, PDGFRα + cMSCs showed slightly increased blood vessel density (<30 µm) (Fig. 6C). In contrast, animals that received hTERT + PDGFRα + cMSCs had a significant increase in blood vessel density compared with controls that received vehicle only (Fig. 6C). No difference in lumen number was observed in the remote non-infarcted areas of all groups (Fig. 6C). These data suggest that hTERT-mediated cardiac repair is partly due to increased angiogenesis within the peri-infarct regions.

### Transplantation of hTERT + PDGFRα + cMSCs into the infarcted heart improves cardiac function and decreases scar size by a telomerase-dependent mechanism

**Figure 7:**
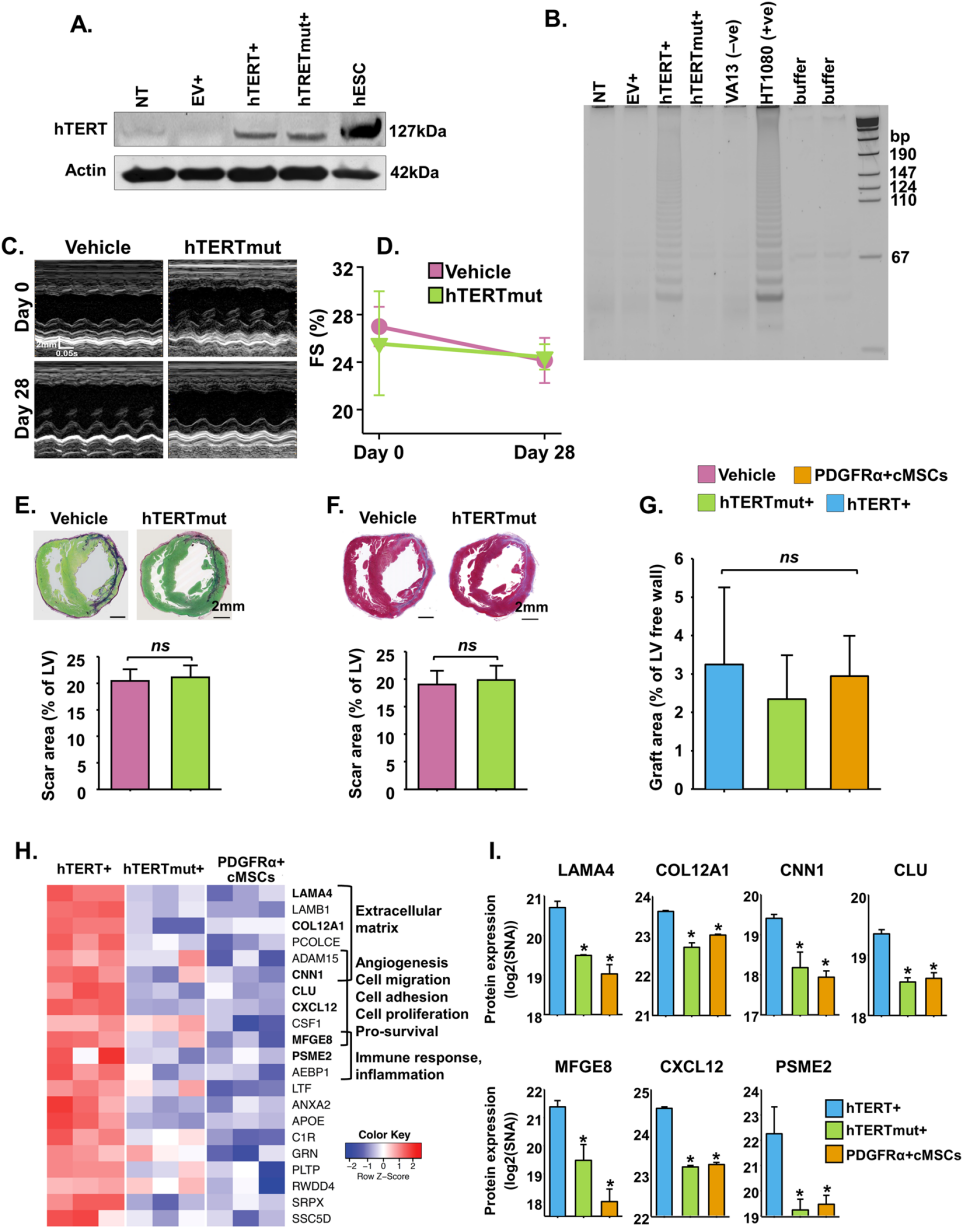
hTERT + PDGFRα + cMSC transplantation improves cardiac function and decreases scar by paracrine effects dependent on telomerase function. Expression of hTERT mutant (hTERTmut) in PDGFRα + cMSCs confirmed by (**A**) Western blot and (**B)** telomeric repeat amplification protocol (TRAP) assay. HT1080 cell line and buffer only were used as telomerase positive and negative controls, respectively. (**C,D**) Representative M-mode images and consecutive echocardiographic data before and four weeks after cell delivery to rats with myocardial infarction. (**E,F**) Fibrotic scar area assessed by picroSirius red and Gomori Trichrome staining. (**G**) Analysis of cell engraftment four days after MI. (**H**) Heat map of differentially expressed proteins from supernatant collected from hTERT + PDGFRα + cMSCs, hTERTmut + PDGFRα + cMSCs or non-transduced PDGFRα + cMSCs. High expression of proteins shown in red and low expression in blue. (**I**) Proteins involved in the regulation of extracellular matrix, angiogenesis, cell proliferation and survival were predominantly found in hTERT + PDGFRα + cMSCs. SNA, sum normalized area; LAMA4, laminin subunit alpha-4; COL12A1, collagen alpha-1(XII) chain; CNN1, calponin-1; CLU, clusterin; MFGE8, milk fat globule-EGF factor 8; CXCL12, C-X-C motif chemokine 12; PSME2, proteasome activator complex subunit 2. N = 5–10 rats/group. Data presented as Mean ± SE; ns, not significant, *p < 0.05, using one-way ANOVA with Holm-Sidak post-test.

hTERT has been shown to have functions both dependent and independent of its catalytic telomerase activity^14,21^. To examine whether hTERT improves cardiac function independently of this catalytic activity, we used a hTERT construct encoding a single amino acid change at position 712 making resultant protein catalytically inactive (D712A hTERT mutant)^28^. Expression of mutant hTERT (hTERTmut) was confirmed by Western blots (Fig. 7A) in the absence of telomerase activity (Fig. 7B). In contrast to the cardiac functional improvements discussed above (Fig. 5), transplantation of hTERTmut + PDGFRα + cMSCs showed no improvement in cardiac function compared to controls (Fig. 7C,D). Similarly, there was no difference in scar size (Fig. 7E,F) nor significant cell engraftment (Fig. 7G).

Since cellular engraftment in the infarcted heart was not observed at 4 weeks after transplantation, we hypothesised the cardiac functional and cellular changes described above are exerted by paracrine mechanisms. To elucidate how hTERT over-expression might impact secreted proteins, we performed mass spectrometry-based untargeted proteomic analysis on the supernatant collected from hTERT + PDGFRα + cMSCs, hTERTmut + PDGFRα + cMSCs and non-transduced PDGFRα + cMSCs. Intriguingly, we observed a distinct pattern of significantly differentially detected proteins (Fig. 7H,I). In particular, hTERT over-expression resulted in increased expression of proteins associated with matrix modulation, angiogenesis, cell proliferation/survival/adhesion and innate immunity function^29–31^ when compared to either hTERTmut + PDGFRα + cMSCs or non-transduced PDGFRα + cMSCs. These results suggest that favourable effects of hTERT + PDGFRα + cMSCs on scar formation and cardiac functional improvement are at least partially dependent on hTERT’s catalytic activity and resultant effects on protein expression in PDGFRα + cMSCs.

## Discussion

Cardiac fibroblasts and progenitor cells are critical for heart function through interaction with cardiomyocytes and extracellular matrix components. These cells play many important roles in response to cardiac injury. We and others have shown that human hearts contain progenitor cells whose functional properties can be harnessed for cardiac repair^15–17,32^. Nevertheless, the exact biological functions of these heterogeneous populations still remain ill-defined. We have previously focused on cardiac progenitor cells in rodents and humans within the cardiac PDGFRα+/CD31^−^ mesenchymal stromal cell population^15–17^. In this study, we have further characterised this mesenchymal fraction and show that hTERT over-expression in PDGFRα + cMSCs induces transcriptional changes favouring increased cell survival, cell differentiation, matrix modulation, and angiogenesis. These qualities are all prudent for cardiac repair therapy. Furthermore, we show hTERT + PDGFRα + cMSCs increase cardiac function after transplantation into the infarcted rat heart. These favourable effects are due to hTERT + PDGFRα + cMSCs manipulation of the infarct microenvironment by a telomerase-dependent mechanism.

The cardiac fibroblast has gained increased attention as an important therapeutic target to treat cardiac diseases^33^. Correspondingly, attention to the molecular phenotype and developmental origins of cardiac fibroblasts has recently increased. *Tcf21* and PDGRFα and PDGRFβ are among the candidate markers that identify cardiac fibroblasts involved with fibrosis after injury^34,35^. Although knowledge is increasing, the vast majority of our understanding about cardiac fibroblasts comes from animal models^36^ and little is known about human cardiac fibroblasts. Our study helps fill this knowledge gap. Recently, in genetic mouse models, cardiac fibroblasts were shown to display cardiogenic gene signatures closely resembling cardiac progenitors and cardiomyocyte precursors^37^. Further, these isolated mouse fibroblasts expressed MSC gene signatures. These results were confirmed in a small selection of human fibroblast samples from patients undergoing procedures for known cardiac diseases^37^. Similarly, we identified cardiogenic factors (*TCF21, TBX18, TBX20, GATA4, GATA6* and *MEF2C)* in PDGFRα + cMSCs. However, in contrast to our findings, Furtado and colleagues^37^ did not show the expression of pluripotency factors (*MYC*, *KLF4* and *SOX4*) that we have identified here. This important difference might be due to our use of cell sorting to select the PDGFRα+/CD90+/CD31^−^ fraction which enriches a broader fibroblast population for progenitor/stem cells. Our present and previous results^15–17^, together with other unbiased transcriptional profiling of the wider cardiac fibroblast pool^37^, suggest that PDGFRα + cMSCs are a subpopulation of cardiac fibroblasts with high cellular plasticity. This raises the possibility of therapeutic modulation of PDGFRα + cMSCs in human patients with cardiac disease. In keeping with reports that *THY1 (CD90)* can identify cardiac progenitors with increased cardiogenic capacity^38^, we found that *CD90* and *TNC* were highly expressed in young, compared to adults, PDGFRα + cMSCs. This suggests an enrichment in the MSC phenotype^38^, and possible increased therapeutic potential of PDGFRα + cMSCs in young, but not aged or diseased, hearts^39^.

The enzyme telomerase is necessary for the synthesis and maintenance of telomere length, which is important for cell proliferation and growth. Although PDGFRα + cMSCs from young hearts proliferated rapidly at early passages but after 90 days this proliferative potential declined (Fig. 1D). It is important to note that, whilst we cannot completely exclude the presence of telomerase or hTERT, it is below the level of detection of the assays used. Consistent with other reports^22^, we found no telomerase activity or hTERT in PDGFRα + cMSCs derived from any hearts (Fig. 2B–D). Without this activity, telomere lengths cannot be replenished resulting in cellular senescence. Senescence will be reached sooner in “adult” samples (which have shorter telomere lengths at the start of culture) as shown in our current (Fig. 1D) and other studies^17,32,40,41^. It is entirely feasible that the longer telomere lengths present in the cells isolated from young hearts provide sufficient proliferative capacity to enable culture for 100 days in the absence of telomerase.

For over a decade, cardiac repair cell therapy has undergone intense research. Despite many stem cell clinical trials in cardiac repair, we are still without any clinically approved strategy. It is increasingly postulated that the poor efficacy of cardiac cell therapy may be remedied by enhancement of candidate cells before delivery into the damaged heart. Such strategies have led to manipulation of stem/progenitor cells using a ‘cocktail’ of cardiopoietic factors^42^, a serine/threonine kinase (PIM1)^6,43^ or hTERT^10,44^ over-expression. In our study, we used lentivirus to stably over-express hTERT in PDGFRα + cMSCs resulting in telomerase activity, concomitant increase in telomere length (Fig. 2) and rejuvenation of an aged phenotype in *in vitro* assays (Figs 2, 3). Consistent with other studies^10,13,44^, this conferred greater cell cycle activity, decreased starvation-induced apoptosis and significantly increased *in vitro* cellular differentiation potential (cardiomyocyte and endothelial cell lineages, Fig. 3).

Our *in vitro* results strongly suggest that forced over-expression of hTERT in donor cells is a promising strategy to enhance cellular therapy. Furthermore, our data shed light on differential cardiac repair effects of aged or diseased cells. In addition to the canonical (telomerase-related) effects of hTERT discussed above, hTERT has increasingly been shown to have non-canonical functions on cell differentiation and stem cell function. In skin, *Tert* over-expression increases stem cell mobilisation and proliferation^45^. This is not dependent on telomere elongation but is mediated by modulation of the Wnt/β-catenin signalling system^20^. Similarly, transcriptional profiling of hTERT + PDGFRα + cMSCs in our study found genes associated with pluripotency and Wnt signalling are significantly enriched in cells from young but not from aged hearts (Fig. 4). In MSCs-derived from adipose tissue^10^ and umbilical cord blood^46^, hTERT increases angiogenesis and myogenic differentiation. Similarly we also found increased angiogenesis and cell differentiation genes in hTERT + PDGFRα + cMSCs. In cancer cell lines, hTERT modulates metalloproteinase expression^47^, and promotes cell adhesion/migration^21^ and inflammation^48^, partly through NF-κB activity. In support of these data showing non-canonical hTERT functions, we also found increased extracellular matrix, cell adhesion, and migration genes in hTERT + PDGFRα + cMSCs. These changes were most apparent in young compared to diseased and adult hTERT + PDGFRα + cMSCs and, strikingly, a significant decrease in NF-κB signalling genes was only observed in young cells. Therefore, our results show, for the first time, the importance of age and disease on receptivity to hTERT-mediated transcriptional change.

Recent pre-clinical studies from multiple groups provide compelling evidence that *Tert* over-expression in cardiac cells via gene therapy or in progenitor cells subsequently transplanted into ischemic murine hind-limbs increases cardiac and skeletal muscular function^10,25,44^. In our study, transplantation of hTERT + PDGFRα + cMSCs in athymic-rats four weeks after induced MI significantly improved LV fractional shortening compared to PDGFRα + cMSCs or vehicle controls. Moreover, hTERT + PDGFRα + cMSC treatment resulted in less scar area and less fibrosis-related αSMA protein in the infarcted myocardium (Figs 5, 6), most likely reflecting decreased myofibroblast activity. Less fibrosis may, in turn, explain the increased cardiac function of hTERT + PDGFRα + cMSC-treated rats. Supporting a role for hTERT in infarct healing, Bar and colleagues^25^ showed that AAV9-Tert gene therapy in the adult mouse heart shows protective effects after MI, improving LV function, decreasing scar formation and increasing mouse survival. Although this study supports our findings, it is interesting to note differences in the models. First, AAV9-Tert gene therapy preferentially results in increased *Tert* expression in cardiomyocytes rather than in progenitor cells and fibroblasts. Second, *Tert* up-regulation required AAV9-Tert treatment two weeks before creation of MI. In our more clinically relevant model, hTERT + PDGFRα + cMSCs delivery four days after MI resulted in similarly increased cardiac repair and improved function. Similar to cardiac AAV9-Tert therapy, delivery of hTERT + PDGFRα + cMSCs in our study resulted in increased cardiomyocyte and non-cardiomyocyte cell cycling (Fig. 6). We also observed significantly increased cardiac vascular density after hTERT + PDGFRα + cMSC delivery, which is in keeping with results of *hTERT + *AT-MSC and endothelial progenitor cell transplantation in hind-limb ischemia models^10,44^. Importantly, in all these preclinical hTERT over-expression studies, no tumorigenicity was observed in the target or remote organs (Supplementary Fig. S8), suggesting safety of future clinical treatment.

Regarding mechanism, the effects of telomerase gene therapy on ageing are thought to be telomerase-dependent and reliant on *Tert* catalytic effects^14^. In our study, we investigated canonical versus non-canonical mechanisms of cardiac functional improvements by using a catalytically inactive hTERT D712A mutant (hTERTmut) construct. Delivery of hTERTmut + PDGFRα + cMSCs failed to increase cardiac function or decrease scar formation after MI, in contrast to the improvements seen with delivery of catalytically active hTERT + PDGFRα + cMSCs. This shows that the overall functional therapeutic effects of hTERT + PDGFRα + cMSC cardiac transplantation are reliant on telomerase activity. Our study shows a temporally-related decrease of transplanted cells (Supplementary Fig. S9A) with no evidence of human cells in the infarcted hearts four weeks after transplantation. Poor engraftment of cardiac progenitor cells (CPCs) and MSCs after transplantation has been reproducibly shown by multiple groups^49,50^. This implies that paracrine mechanisms are essential for the beneficial effects of hTERT + PDGFRα + cMSCs after MI. Further work examining the effects of conditioned media or exosome fractions collected from hTERT and non-hTERT cells on cardiac function and cellular changes *in vivo* are necessary to confirm the beneficial effects of hTERT + PDGFRα + cMSCs through paracrine mechanisms.

Our results place PDGFRα + cMSCs as an important cardiac cell population that could be manipulated to enhance current cardiac disease therapeutics. Enticingly, our results ask questions that could lead to even greater therapeutic impact. First, can PDGFRα + cMSCs be manipulated *in vivo* by gene or protein delivery to effect similar cardiac repair without cell transplantation? Secondly, can the hTERT effect on PDGFRα + cMSC be replicated by other means such as delivery of a suitable small molecule?

**Figure 8:**
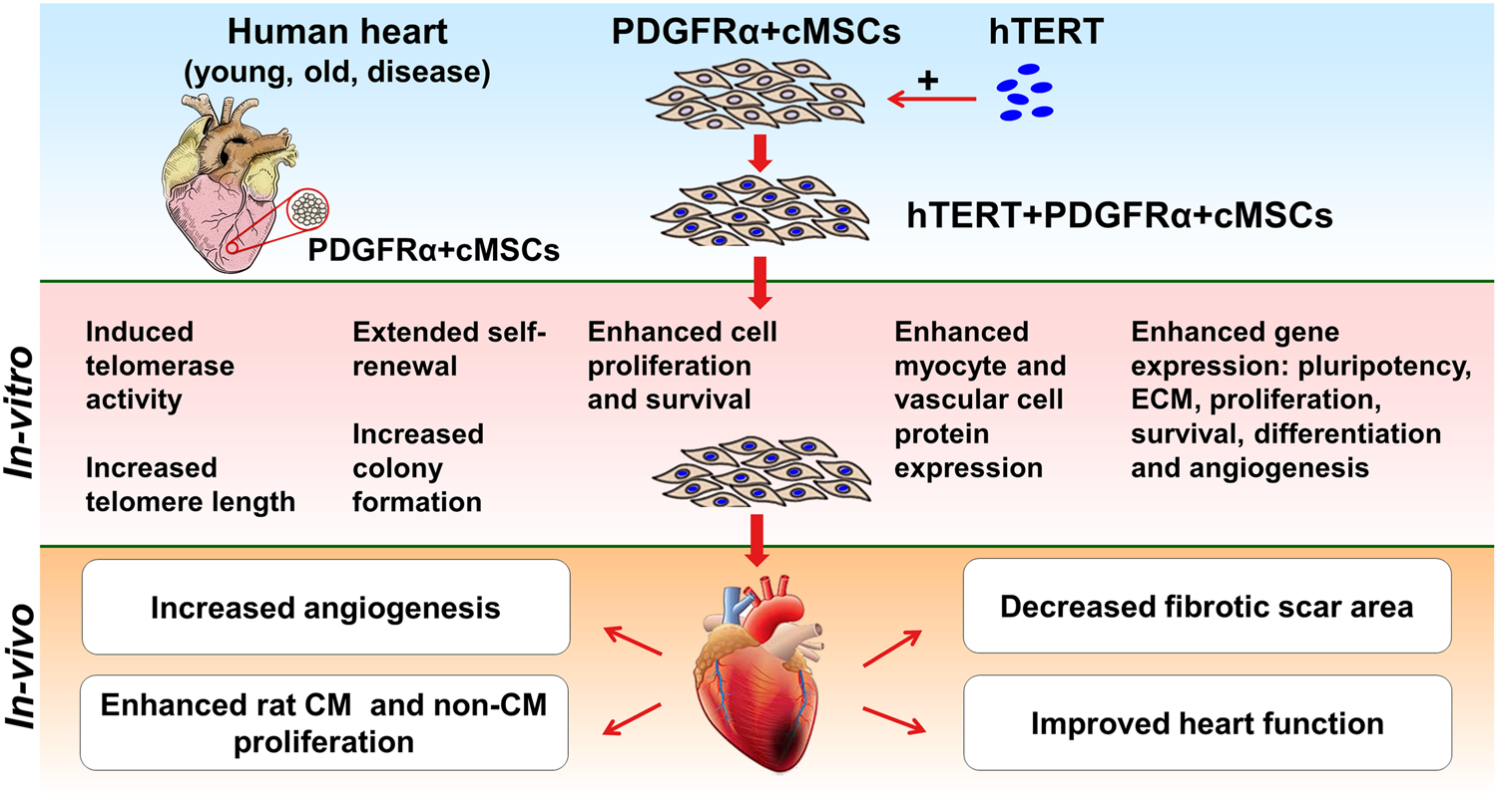
Schematic summary illustration.

In summary, we show that over-expression of hTERT in PDGFRα + cMSCs is an effective strategy to prevent cardiac progenitor cell senescence and to enhance their reparative capabilities (Fig. 8). This provides proof-of-concept for strategies to manipulate these cardiac mesenchymal cells for treatment of patients with large MIs and ensuing HF.

## Methods

### Human samples

Human left ventricle (LV) tissue samples from young (2–10 years) and adult (54–64 years) non-failing donors, as well as from patients 54–64 years undergoing heart transplantation for end-stage idiopathic dilated cardiomyopathy (IDCM) (referred to as “diseased” samples), were provided by the Sydney Heart Bank (Supplementary Table S1). The collection of human tissue from heart transplant patients and donor hearts was approved by the University of Sydney Human Research and Ethics Committee (2012/2814 and 2016/7326), and by St. Vincent’s Hospital (2010 H03/118) and by the Australian Red Cross Blood Service. Heart tissue was collected only when the donor was declared brain-dead by the transplant coordinator. All donors were de-identified and their associated clinical data were stored securely.

### Isolation and culture of human PDGFRα + cardiac mesenchymal stromal cells (PDGFRα + cMSCs)

Human PDGFRα + cMSCs were isolated by explant culture and purified by fluorescence-activated cell sorting as previously described^17^. Briefly, frozen left ventricular samples (~100–200 mg) were removed from liquid nitrogen and quickly thawed by washing twice with ice cold DPBS solution. The tissue was minced into small segments and then placed in 6-well plates coated with 0.1% (wt/vol) gelatin. All explants were cultured in MEMα medium (Sigma-Aldrich) supplemented with 20% FBS (Sigma-Aldrich), 2 mM L-glutamine and 1x penicillin-streptomycin (Sigma-Aldrich), in a 5% CO_2_ humidified incubator at 37 °C. The culture media was replaced every 2–3 days. After 2–3 weeks, the outgrowth of cells was dissociated with trypsin-EDTA (Sigma-Aldrich) and stained with anti-PDGFRα antibody-APC (R&D; 1:10), anti-CD31-PE (R&D; 1:20) and anti-CD90-FITC (BioLegend; 1:20). The cells were then sorted for PDGFRα+/CD90+/CD31^−^ fraction using Influx machine (BD Biosciences), as described previously^17^.

### Over-expression of hTERT in PDGFRα + cMSCs

To generate hTERT stably overexpressing cell lines, HEK293T cells were transfected with pUE-Lenti-hTERT, pUE-Lenti-eGFP or empty pUE-Lenti plasmids and the lentiviral packaging plasmids pMD2 VSV-G, pMDLgp RRE and pRSV REV using calcium phosphate precipitation. The viral supernatants were collected 72 hours after transfection, centrifuged at 3000 rpm for 1–2 hours at 4 °C, and then used to infect PDGFRα + cMSCs from young, adult and diseased samples. The cells were transduced with lentivirus in media containing polybrene (8 µg/ml) overnight and then selected with Blasticidin (10 µg/ml). Non-transduced cells were included as controls. Quantitative reverse transcription PCR (qRT-PCR) and Western blot assays were then performed on transduced and non-transduced cells to determine successful hTERT over-expression.

### RNA-sequencing (RNA-seq) and analysis

RNA-seq was carried out on the sorted cell fraction (PDGFRα+/CD90+/CD31^−^) followed by expansion for 2–3 passages. Total RNA from non-transduced PDGFRα + cMSCs, empty vector or hTERT-transduced PDGFRα + cMSCs was isolated using Isolate II RNA mini kit (Bioline) according to manufacturer’s protocol. RNA was then prepared for sequencing on Illumina HiSeq. 2500 using the Illumina TruSeq Stranded mRNA sample preparation kit Set A (Illumina, CA, USA). Samples were indexed and run twelve per lane, generating ~10 million 50 bp reads per sample.

Raw sequencing reads were analyzed using FastQC to assess the quality of the data and Trimmomatic was used to remove adapter sequences and low-quality reads. The pre-processed reads were then processed using the Falco framework, with STAR 2.5.2a as the aligner and featureCounts 1.5.0 as the read quantification tool. Hg38 reference genome and annotation from GENCODE were used to build index and as annotation for quantification. Read counts were then normalized to read counts per million (rpm) and log2-transformed prior to performing paired differential expression analysis using the limma R package. Genes in hTERT + samples are considered as being significantly differentially expressed if they have a p < 0.05 and an absolute fold change value > 1 compared to the NT samples and that the same genes are not significantly differentially expressed in the EV-NT samples comparisons. Gene set enrichment analysis was performed on the significantly differentially expressed gene list using the g:Profiler R package with the following parameters – False Discovery Rate (FDR) adjusted p < 0.05, gene set with a minimum of 5 genes and maximum of 500 genes to avoid bias due to gene set size, a minimum intersection of 3 genes and a moderate hierarchical filtering to provide higher-level gene set terms. The raw and processed data are accessible from NCBI Gene Expression Omnibus (GSE112297).

### Liquid chromatography-tandem mass spectrometry (LC-MS/MS) and analysis

45 mL conditioned medium was concentrated and the buffer exchanged to 100 mM ammonium bicarbonate to remove all visible dye using Amicon Ultra-15 centrifugal 3 kDa cut-off filter tubes. Urea (6 M) was added and left overnight at 4 °C before reduction with 10 mM DTT, followed by alkylation with 20 mM iodoacetamide. Urea was diluted to 2 M before adding trypsin at a ratio of 1:100 (trypsin: total protein) and digested overnight at 37 °C. The digests were acidified with 1% trifluoroacetic acid before clean-up of peptides on Empore SPE 4 mm/1 mL C18-SD columns. Samples were analyzed by LC-MS/MS using a SCIEX tripleTOF 5600 system coupled to an Eksigent NanoLC Ultra 2D Plus HPLC system. The sample was injected onto a trapping column (PROTECOL C18G, 200 Å, 3 μm, 10 mm × 300 μm) before separation on an analytical column (Acquity UPLC M-class BEHC18 1.7 μm, 300 μm x 150 mm) at a flow rate of 5 μL/min for a total run time of 90 minutes.

The top 30 peptides were subjected to MS/MS analysis and searched against UniProt extracted human-only databases using ProteinPilot software. Raw data were then analysed using Skyline software. Peak areas were summarised by protein ID, with outliers (measurements >2 SD of the median) within each group removed, and log2-transformed prior to running ComBat^51^ to remove batch effect (technical variation) due to variable sample loadings. Differential expression analysis were then performed using limma R package, with proteins considered significantly differentially expressed if they have a FDR-value < 0.05 and an absolute fold change value > 2.

### Cell transplantation studies

Athymic rats (CBH-rnu, 8–12 weeks) were used for experiments. All procedures were conducted in accordance with the guidelines from National Institutes of Health (Guide for the Care and Use of Laboratory Animals). Study protocols were approved by the Western Sydney Local Health District Animal Care and Ethics Committee (AEC4214.02.14). Rats were housed in a facility with 12-hour light and dark phases, and offered ad libitum food and water intake. Rats were anesthetized with isoflurane (2%) and O_2_ (0.2 L/min), orally intubated, and ventilated. To assess the engraftment of human cells in the rat hearts, we conducted two studies. In the first study, the cells were injected into non-MI hearts. The rats were then euthanized at 2, 7 and 14 days after cell injection. In the second study, the cells were injected into MI hearts and hearts were analyzed at 4 and 28 days after cell injections. After specific time points, hearts were harvested and embedded in OCT. The 7 µm thick frozen sections were fixed with 4% PFA, washed, permeabilized and proceed with immunostaining as described above. Human nuclei antibody (Supplementary Table S3) was used to identify human cells in the rat hearts. Images were taken using a slide scanner (Hamamatsu Nanozoomer, Japan) and analyzed using Image J. For myocardial infarct creation, thoracotomy was performed and the left anterior descending coronary artery was ligated for 60 minutes followed by reperfusion. At 4 days after ischemia-reperfusion, the chest was reopened and animals underwent intramyocardial injection of cells or vehicle controls (n = 10 for each group) in a randomized manner. Before cell injection, PDGFRα + cMSCs, hTERT + PDGFRα + cMSCs or hTERTmut + PDGFRα + cMSCs were washed and resuspended in 100 µL RPMI medium. 5 × 10^6^ cells were injected to infarct and border zones using 29 G insulin syringe. For vehicle controls, 100 µL RPMI was administered. At the appropriate time points, the rats were euthanized with CO_2_ exposure and the hearts rapidly were excised for subsequent histological and molecular analysis. The hearts were fixed in formalin followed by paraffin embedding.

### Assessment of cardiac function by echocardiography

Two-dimensional echocardiography was performed at day −4 (before MI), day 0 (pre-cell) and day 28 after cell injections using a Philips Ultrasound System (USA). All functional evaluations were conducted and analyzed by operators blinded to the animal’s treatment group.

### Histology studies

The 5 µm thick paraffin-embedded sections were dewaxed using histoclear followed by series of ethanol washes. Sections were then stained with hematoxylin and eosin, picroSirius red and Masson trichrome. Images were taken using a slide scanner (Hamamatsu Nanozoomer, Japan). Percent fibrotic scar area of the left ventricle (LV) was quantified using Image J software.

Full details of experimental procedures are included in the online supplementary material including analysis of telomerase activity and telomere length, immunostaining, qRT-PCR, long-term growth, colony-forming unit fibroblast, cell apoptosis and differentiation assays.

### Statistical analysis

Data are presented as Means ± Standard Errors of the Mean (SEM) or number (percent). All data were analyzed with SigmaPlot 12.5 software. Statistical comparisons were performed by unpaired Student’s t-test or one-way ANOVA followed by Holm-Sidak post hoc test to adjust for multiple comparisons. *P < *0.05 was considered statistically significant.

## Supplementary information

Supplementary Information
