# Supplementary material for: Enhanced cardiac repair by telomerase reverse transcriptase over-expression in human cardiac mesenchymal stromal cells
Source: Sci Rep. 2019 Jul 22;9:10579. doi: 10.1038/s41598-019-47022-w (PMC6646304; doi:10.1038/s41598-019-47022-w)
Supplement: Supplementary file 1 — Supplementary Information [file 41598_2019_47022_MOESM1_ESM.pdf]

SUPPLEMENTARY INFORMATION

Enhanced cardiac repair by telomerase reverse transcriptase over-expression in human cardiac mesenchymal stromal cells

Thi Yen Loan Le[1,2], Hilda A. Pickett[3], Andrian Yang[4], Joshua W.K. Ho[4], Sujitha Thavapalachandran[1,2,5], Sindhu Igoor[1,2], Sile F. Yang[3], Melad Farraha[1,2,5], Holly K. Voges[6], James E. Hudson[6], Cristobal G. dos Remedios[7], Tracy M. Bryan[8], Eddy Kizana[1,2,5], James J.H. Chong[1,2,5,9] *

[1]Centre for Heart Research, Westmead Institute for Medical Research, The University of Sydney, Westmead, NSW, 2145, Australia.
[2]Department of Cardiology, Westmead Hospital, Westmead, NSW, 2145, Australia.
[3]Telomere Length Regulation Unit, Children's Medical Research Institute, The University of Sydney, Westmead, NSW, 2145, Australia.
[4]Victor Chang Cardiac Research Institute, and St Vincent's Clinical School, University of New South Wales, Darlinghurst, NSW, 2010, Australia.
[5]Sydney Medical School, The University of Sydney, NSW, 2006, Australia.
[6]School of Biomedical Sciences, University of Queensland, St Lucia, Queensland, 4072 Australia.
[7]Department of Anatomy and Histology, School of Medical Sciences, Bosch Institute, The University of Sydney, NSW, 2006, Australia.
[8]Cell Biology Unit, Children's Medical Research Institute, The University of Sydney, Westmead NSW, 2145, Australia.
[9]Victor Chang Cardiac Research Institute, Darlinghurst, NSW, 2010, Australia.

*Correspondence and requests for materials should be addressed to J.J.H.C. (email: james.chong@sydney.edu.au)

Supplementary Methods

Preparation of conditioned media

Non-transduced PDGFRα+cMSCs, empty vector or hTERT-transduced PDGFRα+cMSCs were grown in complete media until they reached 80-85% confluence. The cells were washed with DPBS twice and then changed to serum-free low glucose DMEM media (Sigma-Aldrich). The cells were cultured for 48 hours before obtaining conditioning media. The conditioned media was cleared of cellular debris by centrifugation at 1000 x g for 30 minutes.

Analysis of telomerase activity and telomere length

Telomere length by terminal restriction fragment (TRF) analysis. Terminal restriction fragment (TRF) analysis to determine telomere length was performed as described previously.[1] Briefly, telomeric restriction fragments were prepared by digestion of genomic DNA with HinfI and RsaI, and separated by pulse-field gel electrophoresis (PFGE). Agarose gels were dried at 60°C, denatured, neutralised and hybridised in-gel to γ-^{32}P-ATP-labelled telomere-specific oligonucleotide probe. Gels were subsequently washed and exposed to a PhosphorImager screen overnight, and imaged using a Typhoon™ FLA 9500.

Telomere length analysis by quantitative PCR (qPCR). Telomere length analysis by qPCR was performed as described previously.[2,3]

Telomerase analysis by telomere repeat amplification protocol (TRAP). One million cells were resuspended in 200 µL of CHAPS lysis buffer containing 0.5% CHAPS in 10 mM Tris pH 7.5, 1 mM $MgCl_2$, 1 mM EGTA pH 8.0, 10% glycerol, 1% β-mercaptoethanol and 1 mM phenylmethylsulphonyl fluoride (PMSF). Cell suspensions were then incubated on ice for 30 minutes, followed by centrifugation at 16,000 x g for 20 minutes at 4°C, after which the supernatant was transferred into a fresh tube. PCR reaction mix was prepared for each sample, containing 90 ng M2 primer (5'-AATCCGTCGAGCAGAGTT), 45 ng ACX primer (5'-GCGCGGCTTACCCTTACCCTTACCCTAACC), 1x PCR buffer, 0.2 U Taq polymerase (#11596594001, Roche), and 1 µL of cell lysate, in a final volume of 50 µL. The reaction mix was heated to 30°C for 25 minutes, 95°C for 2 minutes, followed by 30 cycles of 10 seconds at 95°C, 25 seconds at 50°C, and 30 seconds at 72°C. The PCR products were separated by gel electrophoresis on a 10% acrylamide/0.5x TBE gel, in 0.5x TBE at 150 V for 65 minutes. The gel was then stained with SYBR Gold for 15 minutes and imaged using a Typhoon™ FLA 9500.

Karyotyping

Karyotype of non-transduced and hTERT-transduced cells at passage 5 was analysed by G-banding by Trypsin using Giemsa (GTG-banding) to look for signs of malignant transformation. The cells were harvested, fixed and karyotyped at metaphases using a chromosome imaging analyser system.

Immunofluorescence staining

Immunofluorescence staining of the cells was performed as previously described.[3] Briefly, the cells were fixed with 4% paraformaldehyde (PFA), washed, permeabilised and incubated with primary antibodies (Table S3) for 1 hour at room temperature. The cells were then incubated with appropriate fluorochrome-conjugated secondary antibodies, washed and stained with DAPI (1 µg/mL, Sigma-Aldrich). Slides were analysed using an Olympus FV 1000 Confocal Laser Scanning microscope with FV10-ASW 1.7c software (Olympus, Japan).

For BrdU staining, the cells were incorporated with 20 µM BrdU (Sigma-Aldrich) for 2 hours and then fixed with 4% (wt/vol) PFA for 15 min. After that, cells were incubated with 1M HCl for 10 min on ice followed by 2M HCl for 10 min at room temperature before moving them to an incubator for 20 min at 37°C. Immediately after the acid washes, 0.1M sodium borate buffer (pH9.0) was added to the cells for 12 minutes at room temperature. After the cells were washed with PBS/Tween-20 (0.05%), immunofluorescence was performed using anti-BrdU antibody (1:300, BioLegend), as described above.

Flow cytometry analysis

Cells were harvested, washed and stained with conjugated primary antibodies (Table S3) in 2% FBS in PBS for 60 minutes in the dark. Isotype controls were performed concurrently. The analysis was performed using flow cytometry (BD FACS Canto II; BD Biosciences).

Quantitative reverse transcription polymerase chain reaction (qRT-PCR) analysis

Total RNA was extracted using Isolate II RNA mini kit (Bioline). cDNA templates were synthesised from 1 μg RNA using the M-MLV Reverse Transcription System (Promega) with random hexamers (Promega) following manufacturer's instructions. Quantitative PCR (qPCR) was performed on the Biorad CFX384 using SensiFAST SYBR No-ROX Mix (Bioline) with 0.5 μM gene-specific primers listed in Table S2. RT-minus controls were included. PCR conditions were 95°C for 2 minutes and 40 cycles of 95°C for 5s, 60°C for 10s and 72°C for 15s, followed by melt curve analysis. Relative gene expression was calculated using the $2^{-\Delta\Delta Ct}$ method, which was normalised against the housekeeping gene GAPDH.

Western blot

Protein extraction and western blot were performed as previously described.[4] Protein samples were mixed with Laemmli sample buffer containing 10% β-mercaptoethanol prior to boiling at 95°C for 5 minutes. Samples were resolved by SDS-PAGE and transferred onto PVDF membrane (Immobilon-P, Merck Millipore). Membranes were blocked with Odyssey blocking buffer (LICOR Biosciences) and then incubated with hTERT antibody (Rockland, 1:500) overnight at 4°C. Bound antibodies were detected with IRDye 800CW-conjugated secondary antibody (1:5000, LICOR Biosciences). Membranes were scanned using an Odyssey Infrared Imaging system (LICOR Biosciences).

Long-term growth analysis

Long-term growth was performed as previously described.[3,5] Cells were expanded after plating 20,000 cells per T75 flask. Resulting cells were counted and passaged every ten days or when cells reached 80-90% confluence. Cumulative cell numbers were calculated and plotted (log10 scale).

Colony-forming unit fibroblast assay

500 cells were plated into six-well plates (triplicates for each sample). The cells were cultured in completed MEMα media containing 20% FBS for 12 days. The colonies were then fixed with 4% (wt/vol) PFA and stained with 0.05% (wt/vol) crystal violet. Numbers of colonies were quantified according to their size: large (>2 mm), small (<2 mm) and micro (~25-50 cells) colonies.[3,5]

Cell apoptosis assay

The cells were plated in six-well plates below 80% confluence, then 24 hours later were replaced with serum-free medium and incubated for 24 hours. The Annexin V-FITC Apoptosis kit (Abcam) was used to detect cell apoptosis following the manufacturers' instructions. Cells were analysed using flow cytometry (BD FACS Canto II; BD Biosciences).

***In vitro* vascular cell and myocyte differentiation assays**

The cells were seeded onto gelatin-coated glass coverslips in 24-well plates and cultured in complete MEMα media containing 20% FBS. To determine the differentiation potential, the basal medium was removed and specific differentiation medium was added for 14 days with changes every 3-4 days, as described previously.[3,6] For cardiomyocyte differentiation, the cells were co-cultured with neonatal rat ventricular cardiomyocytes (NRVMs). Co-cultures were maintained with M199 medium containing 2% FBS. For endothelial cell differentiation, cells were cultured in IMDM medium (Invitrogen) supplemented with 10 ng/mL VEGF (R&D Systems), 10 ng/mL bFGF and 2% FBS. For smooth muscle cell differentiation, cells were cultured in DMEM-HG medium supplemented with 50 ng/mL PDGF-BB (R&D Systems) and 2% FBS. Cells were fixed after 14 days of differentiation. Immunofluorescence staining was performed as described above. Primary antibodies for cTnT, α-actinin, von Willebrand Factor (vWF) and smooth muscle myosin heavy chain 11 (MYH11) were used (Table S3). Samples were analysed using an Olympus FV 1000 Confocal Laser Scanning

microscope with FV10-ASW 1.7c software (Olympus, Japan). Ten fields per sample were randomly selected for quantification.

Immunostaining of heart sections

Immunostaining was performed on paraffin-embedded sections. Heart sections were deparaffinized using histoclear and series of ethanol. Antigen retrieval was performed using sodium citrate (10 mM) in PBS/Tween-20 (0.05%) followed by blocking with 5% goat serum (Sigma-Aldrich). The sections were incubated with primary antibodies (Table S3) for 1 hour at room temperature followed by secondary antibodies staining. Blood vessels were identified using von Willebrand Factor (vWF). Myofibroblasts were stained using alpha-smooth muscle actin (α-SMA). Cardiomyocytes (CM) and non-CM proliferation were identified using Ki67 and wheat germ agglutinin (WGA) antibodies. Nuclei were identified with DAPI (1 µg/mL, Sigma-Aldrich). Slides were analysed using a slide scanner (Hamamatsu Nanozoomer, Japan) and an Olympus FV 1000 Confocal Laser Scanning microscope with FV10-ASW 1.7c software (Olympus, Japan). Ten fields per sample were selected for quantification.

Active force study

Human pluripotent stem cells. Ethical approval for the use of human embryonic stem cells (hESCs) was obtained from The University of Queensland's Medical Research Ethics Committee (2014000801) and was carried out in accordance with the National Health and Medical Research Council of Australia (NHMRC) regulations. HES3 (WiCell) were maintained as TypLE (ThermoFisher Scientific) passaged cultures using mTeSR-1 (Stem Cell Technologies)/Matrigel (Millipore). Karyotyping and DNA fingerprinting were performed as a quality control.

Cardiac differentiation. Cardiac cells were produced using recently developed protocols where cardiomyocytes and stromal cells are produced in the same differentiation culture;[7-10] multi-cellular cultures are critical for function.[11,12] Based on flow cytometry the cells generated and used for tissue engineering were ~70% α-actinin^{+}/cTnT^{+} hPSC-CMs with the rest being predominantly CD90^{+} stromal cells.[8]

Heart-Dyno hCO fabrication. Heart-dyno culture inserts were fabricated using standard SU-8 photolithography and PDMS molding practices.[7] hCO were fabricated as per.[7] hTERT+GFP+PDGFRα+cMSCs hCO and GFP+PDGFRα+cMSCs hCO were fabricated by incorporating 3,000 cMSCs per hCO (5% total cell number). hCO were cultured in CTRL medium: α-MEM GlutaMAX (ThermoFisher Scientific), 10% fetal bovine serum (FBS) (ThermoFisher Scientific), 200 µM L-ascorbic acid 2-phosphate sesquimagnesium salt hydrate (Sigma) and 1% Penicillin/Streptomycin (ThermoFisher Scientific) with changes every 2-3 days.

Force analysis of hCO in Heart-Dyno. The pole deflection was used to approximate the force of contraction as per.[7] A Leica DMi8 inverted high content Imager was used to capture a 10s time-lapse of each hCO contracting in real time at 37°C. Custom batch processing files were written in Matlab R2013a (Mathworks) to convert the stacked TIFF files to AVI, track the pole movement (using vision.PointTracker), determine the contractile parameters, produce a force-time figure, and export the batch data to an Excel (Microsoft) spreadsheet.

Supplementary Tables

Table S1. Characteristics of patients.

Donor #	SHB Code	Age (years)	Gender	Tissue	Echocardiographic findings
**1**	4.152	4	Male	LV	Normal
**2**	7.050	2	Male	LV	Normal
**3**	3.109	8	Male	LV	Normal
**4**	5.110	9	Male	LV	Normal
**5**	7.044	63	Male	LV	Normal
**6**	7.028	63	Male	LV	Normal
**7**	6.086	60	Male	LV	Normal
**8**	3.163	57	Male	LV	Normal
**9**	4.109	56	Male	LV	n/a
**10**	7.036	56	Male	LV	LVEF 5-20%, LVEDD 78, LVESD 58, FS 15-20%, CO 3.2, CI 1.7
**11**	4.086	58	Male	LV	LV EF 10%, NYHA IV; CO 3.2, CI 1.8; LVEDD 76, LVESD 72
**12**	5.060	54	Male	LV	LVEF 34% NYHA III; CO 4.5, CI 2.3; , LVEDD 77, LVESD 56, FS 35%

NYHA = New York Heart Association; LVEF = left ventricular ejection fraction; n/a= not available; CI = confidence interval; CO = cardiac output; LVEDD = left ventricular end-diastolic diameter; LVESD = left ventricular end-systolic diameter; FS = fractional shortening.

Table S2. Human primers for qRT-PCR.

Gene name	Acronym	Sequence	Product size
Angiopoietin 1	ANGPT1 F	CTGCAGAGAGATGCTCCACA	140bp
	ANGPT1 R	GCCATCTCCGACTTCATGTT	
CD44 molecule (Indian blood group)	CD44 F	CACGTGGAATACACCTGCAA	105bp
	CD44 R	GACAAGTTTTGGTGGCACG	
Collagen Type I Alpha 1	COL1A1 F	CATGTTCAGCTTTGTGGACC	129bp
	COL1A1 R	TTCTGTACGCAGGTGATTGG	
Collagen Type I Alpha 2	COL1A2 F	CTGGTCTCGGTGGGAACTT	138bp
	COL1A2 R	CAGGTCCTTGGAAACCTTGA	
Collagen Type VI Alpha 3	Col6A3 F	TAATTGAATCGAGGAGCCCA	99bp
	Col6A3 R	AAGTGCCGATGTTTCCTCAT	
Collagen Type XII Alpha 1	Col12A1 F	CCCAGGTCCTCCTGGATACTGTGA	108bp
	Col12A1 R	GCAGCACTGGCGACTTAGAAAATGT	
Cyclin Dependent Kinase Inhibitor 1A	CDKN1A F	TACCCTTGTGCCTCGCTCAG	117bp
	CDKN1A R	CGGCGTTTGGAGTGGTAGA	
Discoidin domain receptor 2	DDR2 F	AGGATCCTGCTCCACAGAGA	104bp
	DDR2 R	AGGAACAGCACCAAGAGCAT	
Ecto-5-prime-nucleotidase	CD73 F	ACTTCATGAACGCCCTGC	108bp
	CD73 R	TTGGAAATTTGGCCTCTTTG	
Endoglin	CD105 F	TCCATGTCCTCTTCCTGGAG	109bp
	CD105 R	CTGAGGACCAGAAGCACCTC	
Epicardin	TCF21 F	AGCTACATCGCCCACTTGAG	119bp
	TCF21 R	TTCAGGTCACTCTCGGGTTT	
Epidermal growth factor receptor	EGFR F	GGGCTCTGGAGGAAAAGAAA	93bp
	EGFR R	TCCTCTGGAGGCTGAGAAAA	
Epiregulin	EREG F	AGGAGGATGGAGATGCTCTG	93bp
	EREG R	CACAGTTGTACTGAGGACTGCC	
Fibroblast-specific protein 1	FSP1 F	AGTACTCGGGCAAAGAGGGT	140bp
	FSP1 R	GCTGTCCAAGTTGCTCATCA	
Filamin A, alpha	FLNA F	GTCGCTCTCAGGAACAGCAG	92bp
	FLNA R	AGGGGACGGCCCTTTAAT	
GATA-binding factor 2	GATA2 F	CCTCCAGCTTCACCCCTAA	135bp
	GATA2 R	CACAGGCATTGCACAGGTAGT	
GATA-binding factor 4	GATA4 F	AGGCCTCTTGCAATGCGGAA	112bp
	GATA4 R	CGGGAGGAAGGCTCTCACTG	
GATA-binding factor 6	GATA6 F	CAGCAAAAATACTTCCCCCA	107bp
	GATA6 R	ACTTGAGCTCGCTGTTCTCG	
Glyceraldehyde 3-phosphate dehydrogenase	GAPDH F	ACCCACTCCTCCACCTTTG	178bp
	GAPDH R	CTCTTGTGCTCTTGCTGGG	
Heme Oxygenase 1	HMOX1 F	AGCTGCTGACCCATGACAC	115bp
	HMOX1 R	GGCTTCCCTCTGGGAGTCT	

Insulin-like growth factor 2 mRNA-binding protein 3	IGF2BP3 F	ATCCCGCCTCATTTACAGTG	100bp
	IGF2BP3 R	CTGCAGTTTCCGAGTCAGTG	
Insulin-like growth factor-binding protein 3	IGFBP3 F	CTCTGCGTCAACGCTAGTGC	95bp
	IGFBP3 R	CGGTCTTCCTCCGACTCAC	
Insulin-like growth factor-binding protein 5	IGFBP5 F	TTTGCCTCAACGAAAAGAGC	118bp
	IGFBP5 R	CGGAAGATCTTGGGGGAGTA	
Integrin beta-1	CD29 F	ATGTGAATGCCAAAGCGAAG	105bp
	CD29 R	CTACCAACACGCCCTTCATT	
Integrin subunit alpha-5 (ITGA5)	CD49e F	CTCAGTGGAGTTTTACCGGC	108bp
	CD49e R	AGGTAGACAGCACCACCCTG	
Integrin subunit alpha V (ITGAV)	CD51 F	GTGACTGGTCTTCTACCCGC	110bp
	CD51 R	TCCAAACCACTGATGGGACT	
Kruppel-like factor 4	KLF4 F	GAGTTCCCATCTCAAGGCAC	130bp
	KLF4 R	CCCCGTGTGTTTACGGTAGT	
Laminin subunit alpha-4	LAMA4 F	CGACAACGCTTTTCCTTTTG	146bp
	LAMA4 R	CCGACAGGGTGTGAAAGAAT	
leucine-rich repeat-containing receptor5	LGR5 F	CAGCGTCTTCACCTCCTACC	109bp
	LGR5 R	GTTTCCCGCAAGACGTAACT	
Matrix metalloproteinase-2	MMP2 F	TTGCTGGAGACAAATTCTGG	148bp
	MMP2 R	AAGAAGTAGCTGTGACCGCC	
Matrix metalloproteinase-19	MMP19 F	TCAAGCCAGAAGATATCACCG	92bp
	MMP19 R	GGCCCTTGTGGCATCAT	
Mitogen-activated protein kinase 12	MAPK12 F	TGTATCGGCCCTTCCAGTC	110bp
	MAPK12 R	AGTGAATACGTCCAGCAGCC	
Mitogen-activated protein kinase 13	MAPK13 F	ATTGGGCTCCTGGATGTCTT	122bp
	MAPK13 R	TCCTCACTGAACTCCATCCC	
Myocyte-specific enhancer factor 2C	MEF2C F	CAGTGCAGGGAACGGGTATG	174bp
	MEF2C R	GGCATCGTATTCTTGCTGCC	
Periostin	POSTN F	AGTTTGTTCGTGGTAGCACCT	139bp
	POSTN R	AGTGTGGGTCCTTCAGTTTTGA	
Platelet derived growth factor receptor alpha (CD140a)	PDGFRA F	AACCGTGTATAAGTCAGGGGA	126bp
	PDGFRA R	ATTTCTTCCAGCATTGTGAT	
Platelet derived growth factor receptor beta (CD140b)	PDGFRB F	CGTCAAGATGCTTAAATCCACAGC	146bp
	PDGFRB R	TGATGATATAGATGGGTCCTCCTTTG	
Platelet endothelial cell adhesion molecule 1	PECAM1 F	CCTTCTGCTCTGTTCAAGCC	110bp
	PECAM1 R	GGGTCAGGTTCTTCCCATTT	
Serine/threonine-protein kinase	PIM2 F	GGTGGCCATCAAAGTGATTC	93bp
	PIM2 R	CATAGCAGTGCGACTTCGAG	
T-box transcription factor	TBX20 F	TTTTGCCAAAGGATTCCGGG	111bp
	TBX20 R	CCGTAGGTACGGATGGGTGA	
Human telomerase reverse transcriptase (TERT)	hTERT F	CGGAAGAGTGTCTGGAGCAA	275bp
	hTERT R	TCGTAGTTGAGCACGCTGAACAG	
Human telomerase RNA	hTR F	CTAACCCTAACTGAGAAGGGCGTA	154bp

component (TERC)	hTR R	GGCGAACGGGCCAGCAGCTGACATT	
Tenascin C	TNC F	AAGTGAACCTGTCTCAGGGTCATT	68bp
	TNC R	TGGCTGTCACCAGGCCAGATG	
Transcription factor SOX-4	SOX4 F	TCGCTGTCGGGTCTCTAGTT	106bp
	SOX4 R	AATGTATGTTTCCCCCTCCC	
Transforming growth factor beta 1	TGFB1 F	CCAAAGGAAAATCTGTGGCA	107bp
	TGFB1 R	TTGAGAGTGGTAGGGCTGCT	
Transforming growth factor, beta receptor III	TGFBR3 F	AGCTCCTGTTTAGCCACTGC	103bp
	TGFBR3 R	CAGTGAAGCTCTCCATCAAGG	
Thy-1 cell surface antigen	CD90 F	CGGAAGACCCCAGTCCA	147bp
	CD90 R	ACGAAGGCTCTGGTCCACTA	
Vimentin	VIM F	CTTCAGAGAGAGGAAGCCGA	97bp
	VIM R	ATTCCACTTTGCGTTCAAGG	

Table S3. Antibodies for FACS and immunofluorescence (IF).

Antibody	Conjugation	Concentration	Isotype	Catalogue number	Company	Use
CD140a/PDGFRa	APC	1:10	IgG1	FAB1264A	R&D Systems	FACS
CD140b/PDGFRb	PE	1:20	IgG1	323606	BioLegend	FACS
CD90 (Thy1)	FITC	1:20	IgG1	328108	BioLegend	FACS
c-Kit (clone 104D2)	N/A	1:20	IgG1	ab111033	Abcam	FACS
CD31/PECAM1	PE	1:20	IgG1	12-0319	eBioscience	FACS
CD34	Alexa Fluor 488		IgG1	343517	eBioscience	FACS
CD45	FITC	1:20			BioLegend	FACS
Annexin V	FITC	10uL per assay		Kit	Abcam	FACS
Vimentin	N/A	1:500			Abcam	IF (cell)
CD90	N/A	1:200	IgG	328102	328102	IF (cell)
PDGFRa	N/A	1:200		A61219	Abcam	IF (cell)
Ki67	N/A	1:200	IgG1	350502	BioLegend	IF (cell)
BrdU	N/A	1:300	IgG1	317902	BioLegend	IF (cell)
CD31	N/A	1:100	IgG	NOVNB100-2284	Novus Biologicals	IF (cell)
Smooth muscle Myosin heavy chain 11 (MYH11)	N/A	1:200	IgG	ab53219	Abcam	IF (cell)
a-actinin	N/A	1:1000	IgG1	A-7811	Sigma	IF (cell)
Cardiac Troponin T (CT3 clone)	N/A	1:1000	MIgG2a	CT3	DSHB	IF (cell)
Human Nuclei	N/A	1:100	IgG1	MAB1281	Merck Millipore	IF (cell)
Smooth muscle Actin (αSMA)	N/A		IgG2a	M085129	Agilent Technologies	IF (tissue)
Ki67	N/A	1:200	IgG	ab15580	Abcam	IF (tissue)
α-actinin	N/A	1:100	IgG	ab68167	Abcam	IF (tissue)
Von Willebrand Factor (vWF)	N/A	1:500		A008229-2	Agilent Technologies	IF (tissue)

Supplementary Figures and Figure Legends

Figure S1.

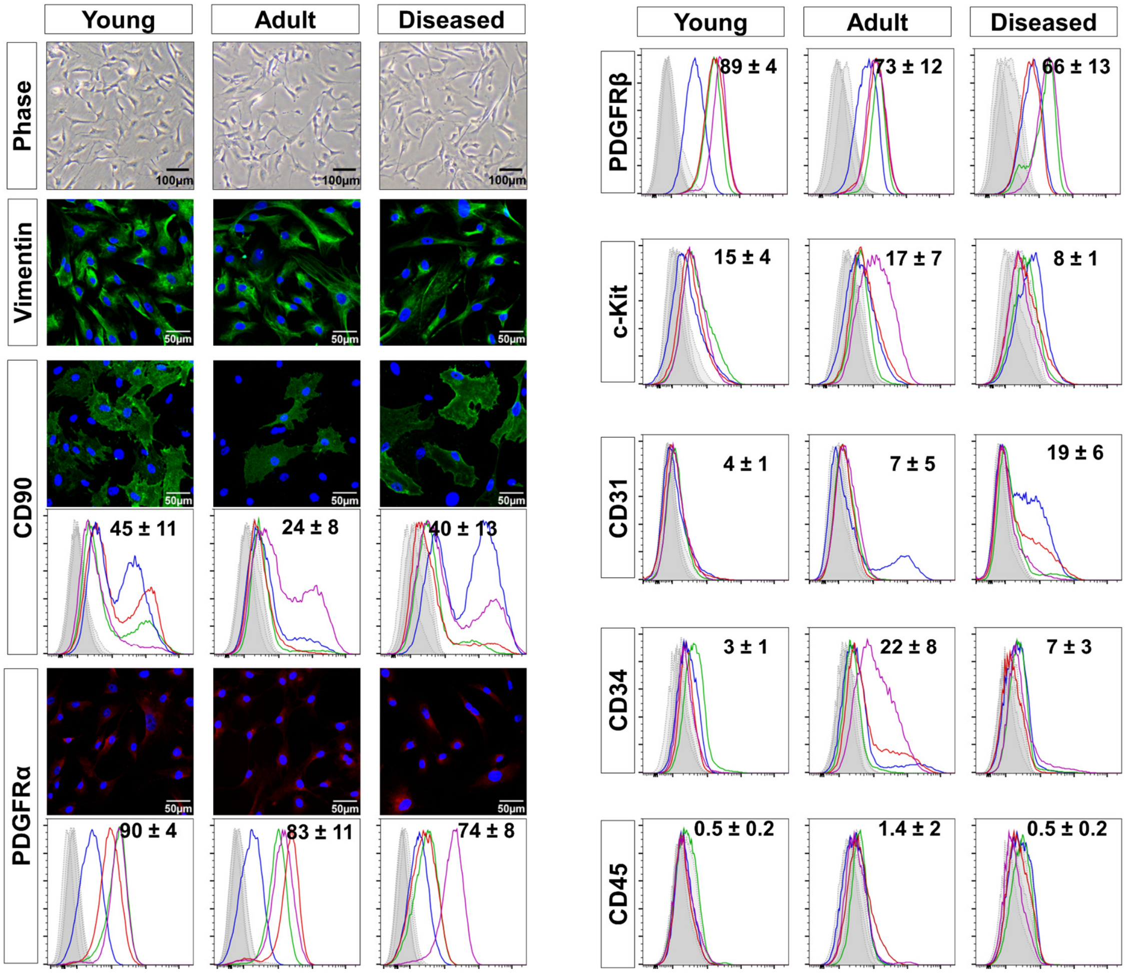

Figure S1. Characterisation of cardiac cells isolated from young, adult and diseased hearts. Representative images and quantification of immunofluorescence and flow cytometric analysis of the cardiac explant-derived cells from young, adult and diseased hearts. Cells highly express fibroblast and cardiac mesenchymal stem cell markers including Vimentin, CD90, PDGFRα, and PDGFRβ. Note minimal expression of c-Kit, CD31 and CD34. No expression of CD45. Data presented as Mean ± SEM; N = 4 patient samples/group.

Figure S2.

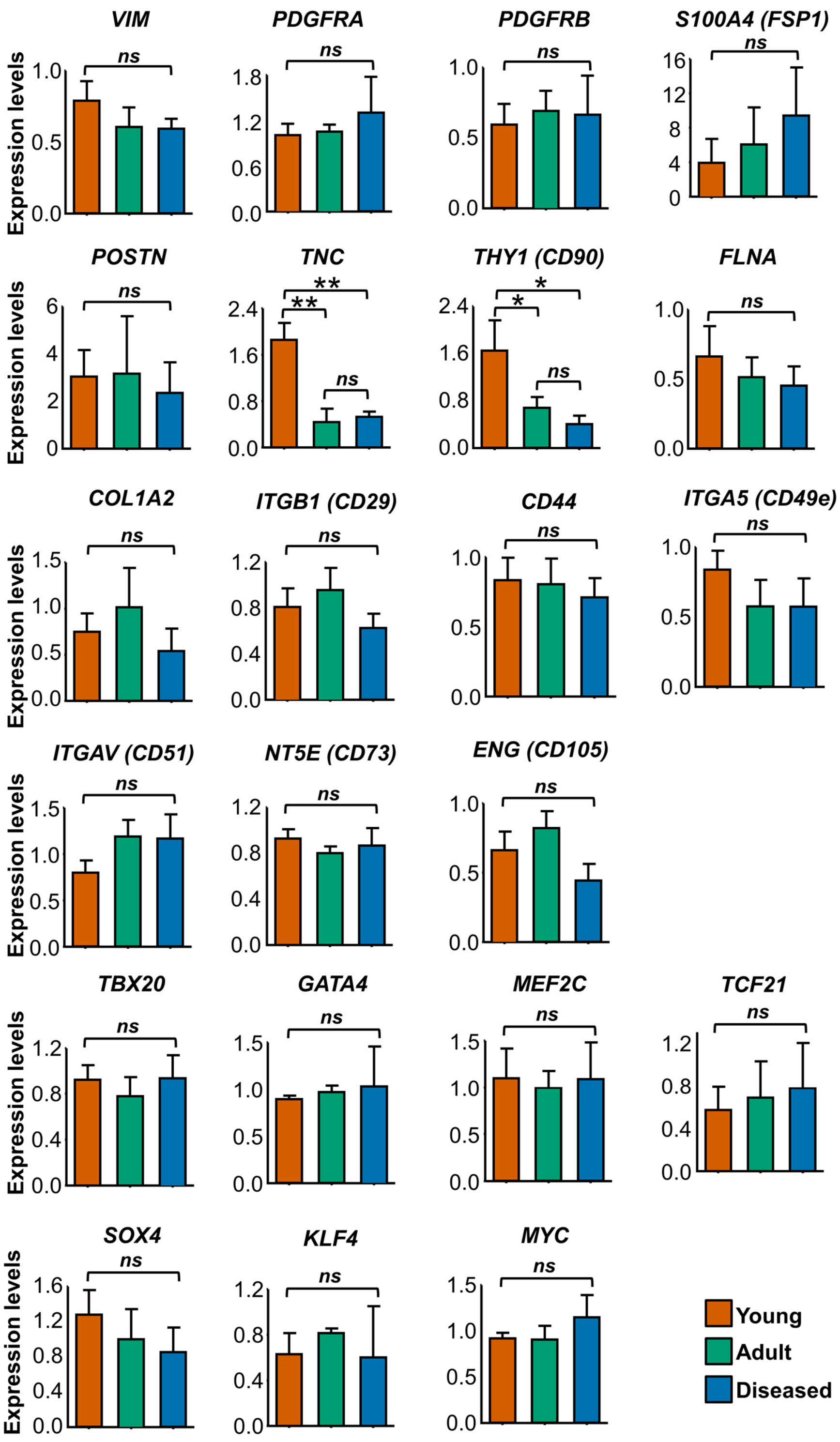

Figure S2. Validation of RNA-sequencing results (see Fig. 1). Expression of fibroblast, mesenchymal stem cell, cardiogenic and pluripotency genes through qRT-PCR.

Figure S3.

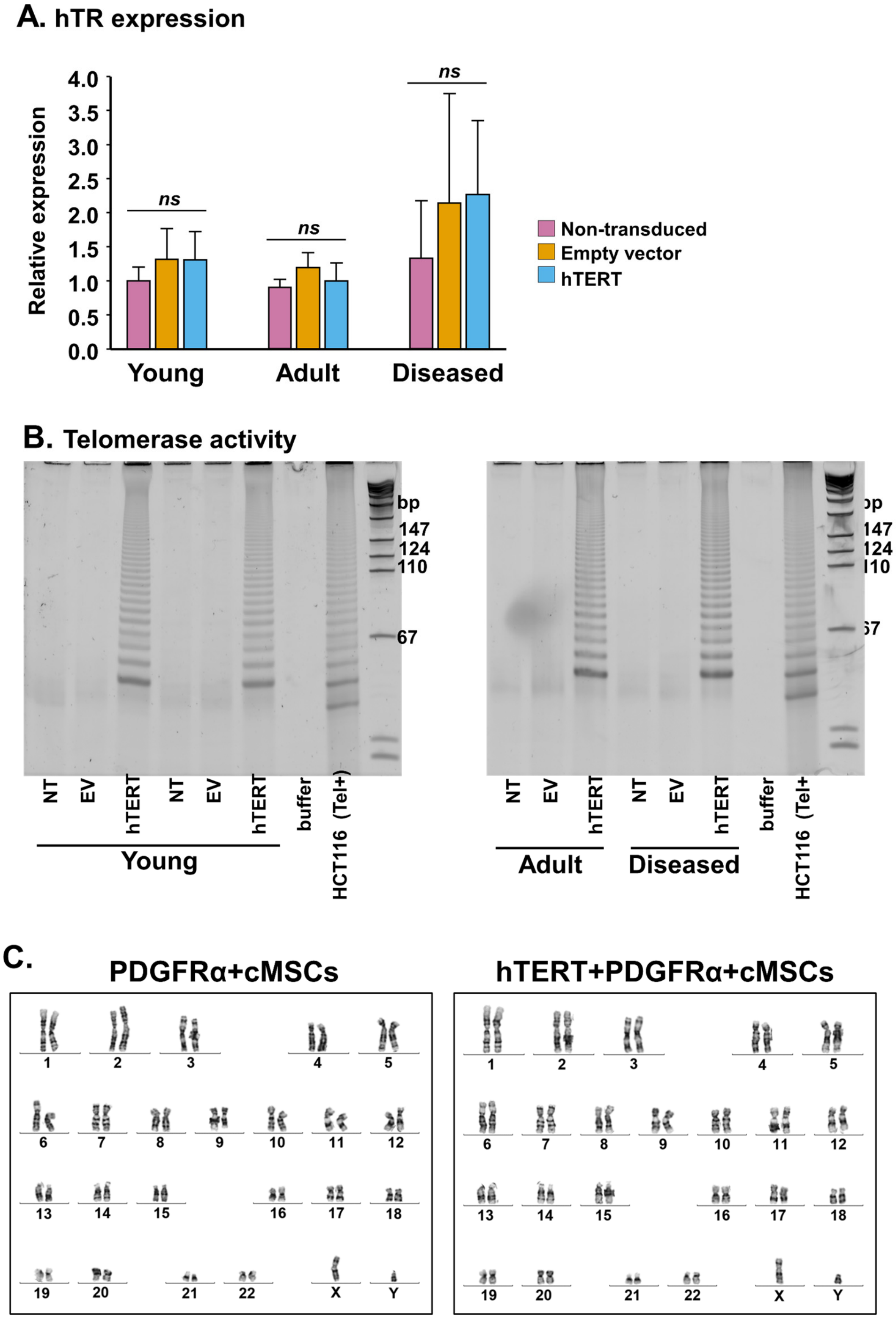

Figure S3. Telomerase RNA component (hTR) and telomerase activity analysis of non-transduced and hTERT over-expressing PDGFRα+cMSCs. (A) hTR analysis by qPCR. Data presented as Mean ± SEM; N = 4 patient samples/group; ns, not significant, using one-way ANOVA with Holm-Sidak post-test. **(B)** Telomerase activity by Telomeric Repeat Amplification Protocol (TRAP) shows telomerase activity detected in hTERT transduced PDGFRα+cMSCs from young, adult and diseased hearts. HTC116 and HT1080 cell lines were used as positive telomerase controls. NT= non-transduced; EV= empty vector. **(C)** Karyotyping of hTERT+PDGFRα+cMSCs shows normal phenotype. See also Figure 2.

Figure S4.

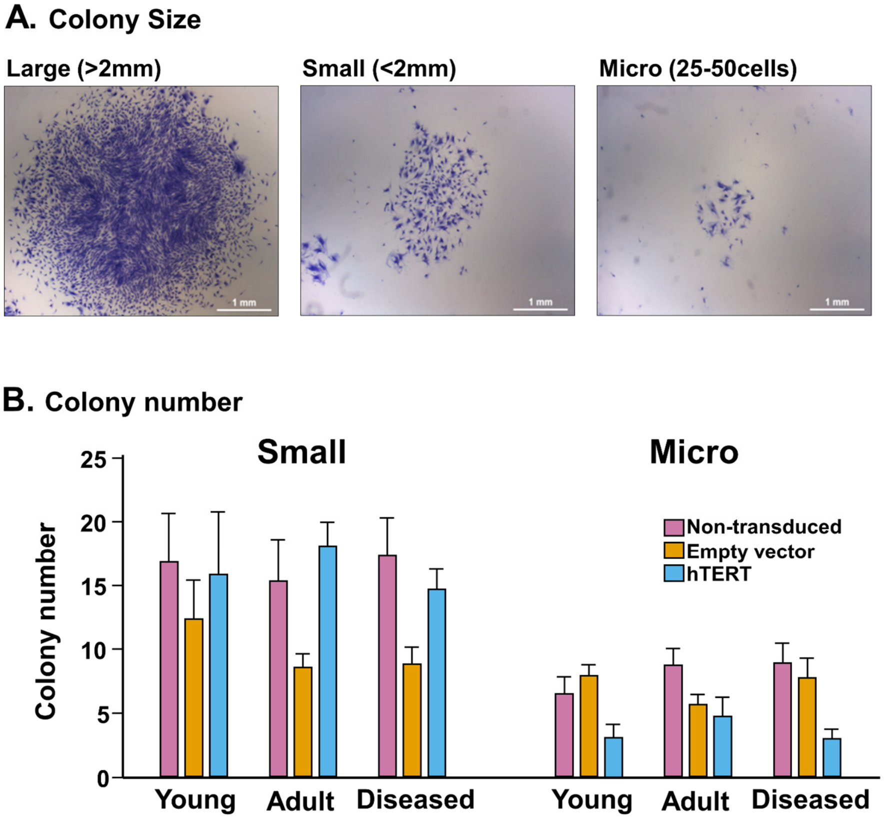

Figure S4. Colony-forming assays of PDGFRα+cMSCs. (A) Cell colonies were stained with crystal violet dye and counted after colony-forming unit fibroblast assays. **(B)** Representative distribution of colony number and size is shown (small and micro colonies; refer to Figure 2 for large colony). Data presented as Mean ± SEM; N = 4 patient samples/group. See also Figure 2.

Figure S5.

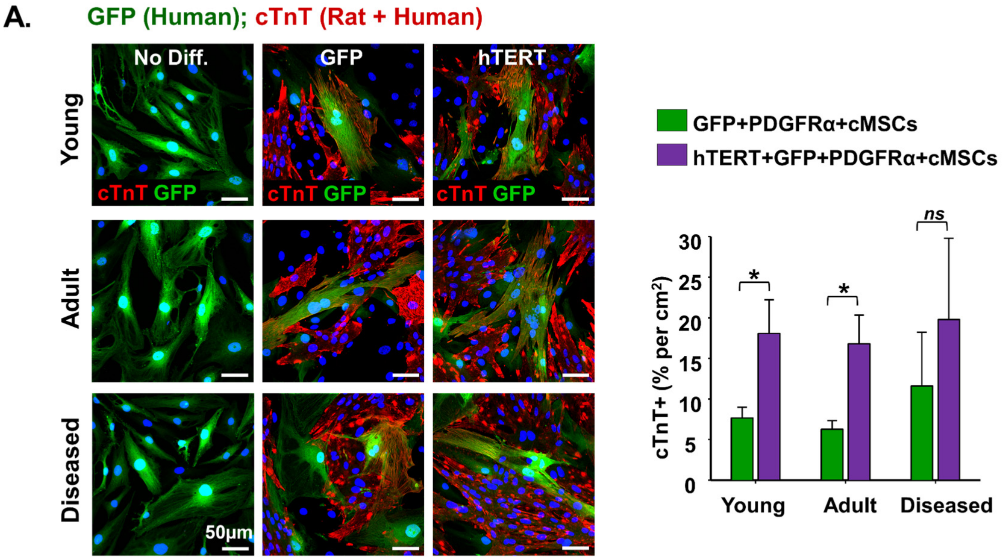

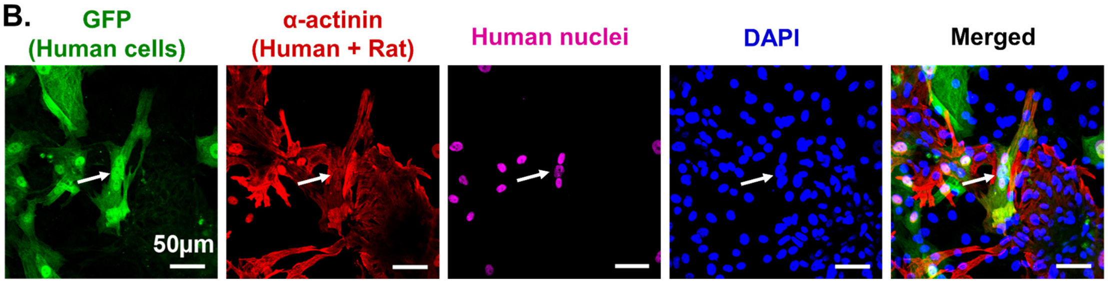

Figure S5. hTERT over-expression enhances PDGFRα+cMSC *in vitro* differentiation to cardiomyocyte lineages. (A) Representative images of non-transduced and hTERT over-expressing PDGFRα+cMSCs co-cultured with neonatal rat ventricular myocytes (NRVMs) for 14 days then stained for cardiac troponin-T (cTnT). Green = GFP (Human cells); Red = cTnT (Human cells and NRVMs). N = 4 patient samples/group. Data presented as Mean ± SEM; ns, not significant; $*p<0.05$, using unpaired Student's t-test. **(B)** Cells from cardiomyocyte differentiation were co-stained with human nuclei antibody to exclude cell fusion. See also Figure 3.

Figure S6.

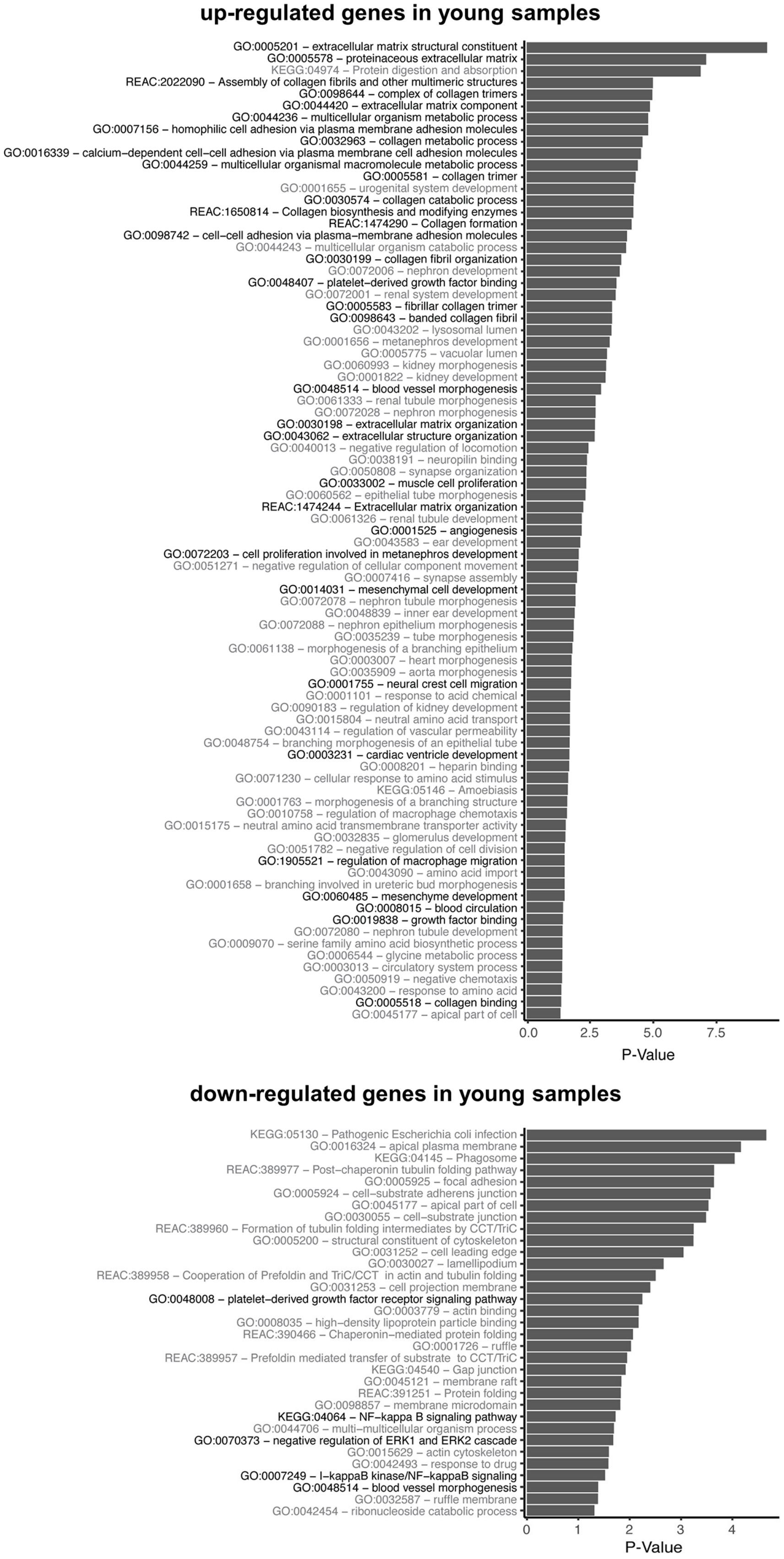
up-regulated genes in young samples
GO:0005201 – extracellular matrix structural constituent
GO:0005578 – proteinaceous extracellular matrix
KEGG:04974 – Protein digestion and absorption
REAC:2022090 – Assembly of collagen fibrils and other multimeric structures
GO:0098644 – complex of collagen trimers
GO:0044420 – extracellular matrix component
GO:0044236 – multicellular organism metabolic process
GO:0007156 – homophilic cell adhesion via plasma membrane adhesion molecules
GO:0032963 – collagen metabolic process
GO:0016339 – calcium–dependent cell–cell adhesion via plasma membrane cell adhesion molecules
GO:0044259 – multicellular organismal macromolecule metabolic process
GO:0005581 – collagen trimer
GO:0001655 – urogenital system development
GO:0030574 – collagen catabolic process
REAC:1650814 – Collagen biosynthesis and modifying enzymes
REAC:1474290 – Collagen formation
GO:0098742 – cell–cell adhesion via plasma–membrane adhesion molecules
GO:0044243 – multicellular organism catabolic process
GO:0030199 – collagen fibril organization
GO:0072006 – nephron development
GO:0048407 – platelet–derived growth factor binding
GO:0072001 – renal system development
GO:0005583 – fibrillar collagen trimer
GO:0098643 – banded collagen fibril
GO:0043202 – lysosomal lumen
GO:0001656 – metanephros development
GO:0005775 – vacuolar lumen
GO:0060993 – kidney morphogenesis
GO:0001822 – kidney development
GO:0048514 – blood vessel morphogenesis
GO:0061333 – renal tubule morphogenesis
GO:0072028 – nephron morphogenesis
GO:0030198 – extracellular matrix organization
GO:0043062 – extracellular structure organization
GO:0040013 – negative regulation of locomotion
GO:0038191 – neuropilin binding
GO:0050808 – synapse organization
GO:0033002 – muscle cell proliferation
GO:0060562 – epithelial tube morphogenesis
REAC:1474244 – Extracellular matrix organization
GO:0061326 – renal tubule development
GO:0001525 – angiogenesis
GO:0043583 – ear development
GO:0072203 – cell proliferation involved in metanephros development
GO:0051271 – negative regulation of cellular component movement
GO:0007416 – synapse assembly
GO:0014031 – mesenchymal cell development
GO:0072078 – nephron tubule morphogenesis
GO:0048839 – inner ear development
GO:0072088 – nephron epithelium morphogenesis
GO:0035239 – tube morphogenesis
GO:0061138 – morphogenesis of a branching epithelium
GO:0003007 – heart morphogenesis
GO:0035909 – aorta morphogenesis
GO:0001755 – neural crest cell migration
GO:0001101 – response to acid chemical
GO:0090183 – regulation of kidney development
GO:0015804 – neutral amino acid transport
GO:0043114 – regulation of vascular permeability
GO:0048754 – branching morphogenesis of an epithelial tube
GO:0003231 – cardiac ventricle development
GO:0008201 – heparin binding
GO:0071230 – cellular response to amino acid stimulus
KEGG:05146 – Amoebiasis
GO:0001763 – morphogenesis of a branching structure
GO:0010758 – regulation of macrophage chemotaxis
GO:0015175 – neutral amino acid transmembrane transporter activity
GO:0032835 – glomerulus development
GO:0051782 – negative regulation of cell division
GO:1905521 – regulation of macrophage migration
GO:0043090 – amino acid import
GO:0001658 – branching involved in ureteric bud morphogenesis
GO:0060485 – mesenchyme development
GO:0008015 – blood circulation
GO:0019838 – growth factor binding
GO:0072080 – nephron tubule development
GO:0009070 – serine family amino acid biosynthetic process
GO:0006544 – glycine metabolic process
GO:0003013 – circulatory system process
GO:0050919 – negative chemotaxis
GO:0043200 – response to amino acid
GO:0005518 – collagen binding
GO:0045177 – apical part of cell
0.0
2.5
5.0
7.5
P-Value
down-regulated genes in young samples
KEGG:05130 – Pathogenic Escherichia coli infection
GO:0016324 – apical plasma membrane
KEGG:04145 – Phagosome
REAC:389977 – Post–chaperonin tubulin folding pathway
GO:0005925 – focal adhesion
GO:0005924 – cell–substrate adherens junction
GO:0045177 – apical part of cell
GO:0030055 – cell–substrate junction
REAC:389960 – Formation of tubulin folding intermediates by CCT/TriC
GO:0005200 – structural constituent of cytoskeleton
GO:0031252 – cell leading edge
GO:0030027 – lamellipodium
REAC:389958 – Cooperation of Prefoldin and TriC/CCT in actin and tubulin folding
GO:0031253 – cell projection membrane
GO:0048008 – platelet–derived growth factor receptor signaling pathway
GO:0003779 – actin binding
GO:0008035 – high–density lipoprotein particle binding
REAC:390466 – Chaperonin–mediated protein folding
GO:0001726 – ruffle
REAC:389957 – Prefoldin mediated transfer of substrate to CCT/TriC
KEGG:04540 – Gap junction
GO:0045121 – membrane raft
REAC:391251 – Protein folding
GO:0098857 – membrane microdomain
KEGG:04064 – NF–kappa B signaling pathway
GO:0044706 – multi–multicellular organism process
GO:0070373 – negative regulation of ERK1 and ERK2 cascade
GO:0015629 – actin cytoskeleton
GO:0042493 – response to drug
GO:0007249 – I–kappaB kinase/NF–kappaB signaling
GO:0048514 – blood vessel morphogenesis
GO:0032587 – ruffle membrane
GO:0042454 – ribonucleoside catabolic process
0
1
2
3
4
P-Value

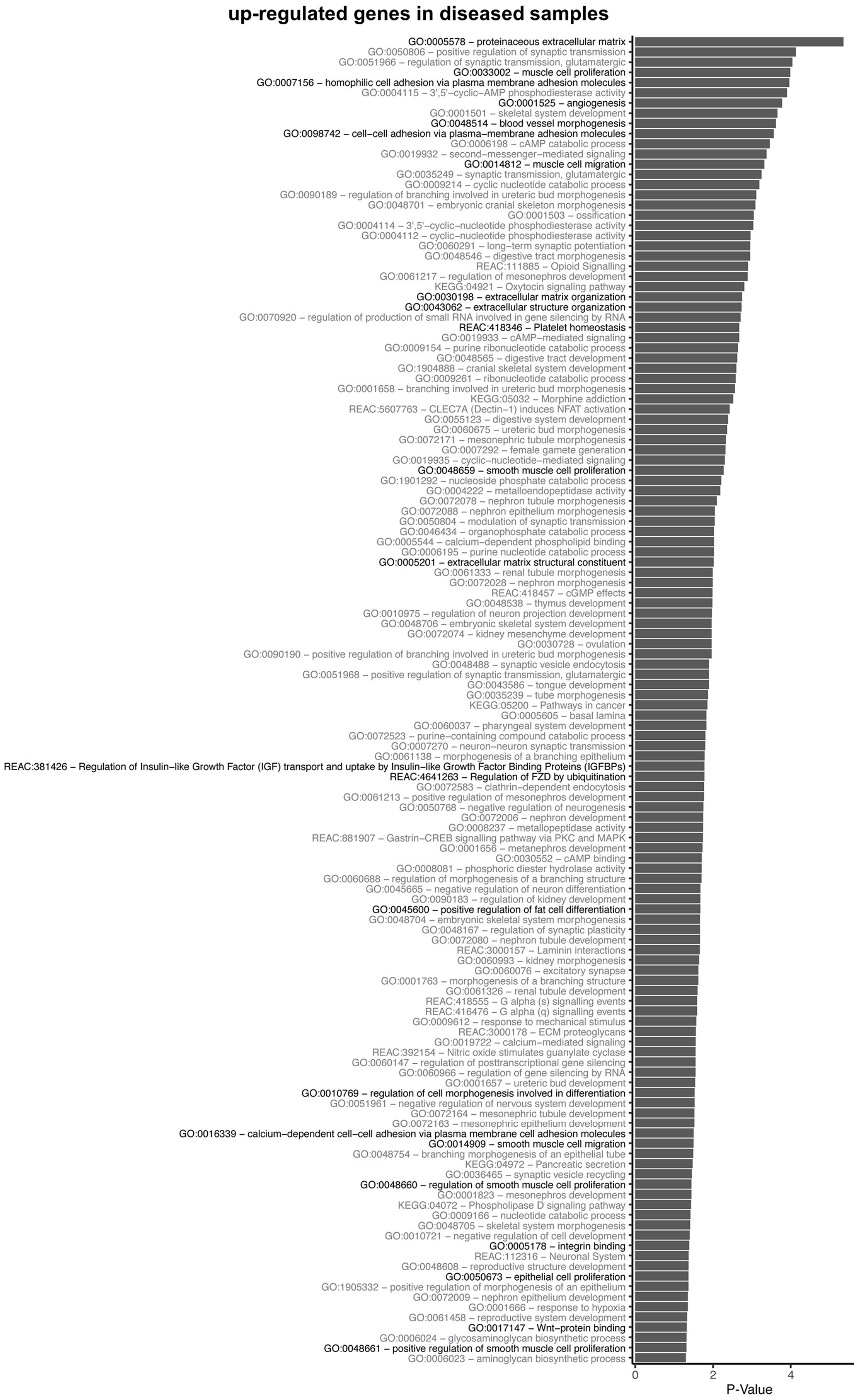
up-regulated genes in diseased samples
GO:0005578 – proteinaceous extracellular matrix
GO:0050806 – positive regulation of synaptic transmission
GO:0051966 – regulation of synaptic transmission, glutamatergic
GO:0033002 – muscle cell proliferation
GO:0007156 – homophilic cell adhesion via plasma membrane adhesion molecules
GO:0004115 – 3',5'–cyclic–AMP phosphodiesterase activity
GO:0001525 – angiogenesis
GO:0001501 – skeletal system development
GO:0048514 – blood vessel morphogenesis
GO:0098742 – cell–cell adhesion via plasma–membrane adhesion molecules
GO:0006198 – cAMP catabolic process
GO:0019932 – second–messenger–mediated signaling
GO:0014812 – muscle cell migration
GO:0035249 – synaptic transmission, glutamatergic
GO:0009214 – cyclic nucleotide catabolic process
GO:0090189 – regulation of branching involved in ureteric bud morphogenesis
GO:0048701 – embryonic cranial skeleton morphogenesis
GO:0001503 – ossification
GO:0004114 – 3',5'–cyclic–nucleotide phosphodiesterase activity
GO:0004112 – cyclic–nucleotide phosphodiesterase activity
GO:0060291 – long–term synaptic potentiation
GO:0048546 – digestive tract morphogenesis
REAC:111885 – Opioid Signalling
GO:0061217 – regulation of mesonephros development
KEGG:04921 – Oxytocin signaling pathway
GO:0030198 – extracellular matrix organization
GO:0043062 – extracellular structure organization
GO:0070920 – regulation of production of small RNA involved in gene silencing by RNA
REAC:418346 – Platelet homeostasis
GO:0019933 – cAMP–mediated signaling
GO:0009154 – purine ribonucleotide catabolic process
GO:0048565 – digestive tract development
GO:1904888 – cranial skeletal system development
GO:0009261 – ribonucleotide catabolic process
GO:0001658 – branching involved in ureteric bud morphogenesis
KEGG:05032 – Morphine addiction
REAC:5607763 – CLEC7A (Dectin–1) induces NFAT activation
GO:0055123 – digestive system development
GO:0060675 – ureteric bud morphogenesis
GO:0072171 – mesonephric tubule morphogenesis
GO:0007292 – female gamete generation
GO:0019935 – cyclic–nucleotide–mediated signaling
GO:0048659 – smooth muscle cell proliferation
GO:1901292 – nucleoside phosphate catabolic process
GO:0004222 – metalloendopeptidase activity
GO:0072078 – nephron tubule morphogenesis
GO:0072088 – nephron epithelium morphogenesis
GO:0050804 – modulation of synaptic transmission
GO:0046434 – organophosphate catabolic process
GO:0005544 – calcium–dependent phospholipid binding
GO:0006195 – purine nucleotide catabolic process
GO:0005201 – extracellular matrix structural constituent
GO:0061333 – renal tubule morphogenesis
GO:0072028 – nephron morphogenesis
REAC:418457 – cGMP effects
GO:0048538 – thymus development
GO:0010975 – regulation of neuron projection development
GO:0048706 – embryonic skeletal system development
GO:0072074 – kidney mesenchyme development
GO:0030728 – ovulation
GO:0090190 – positive regulation of branching involved in ureteric bud morphogenesis
GO:0048488 – synaptic vesicle endocytosis
GO:0051968 – positive regulation of synaptic transmission, glutamatergic
GO:0043586 – tongue development
GO:0035239 – tube morphogenesis
KEGG:05200 – Pathways in cancer
GO:0005605 – basal lamina
GO:0060037 – pharyngeal system development
GO:0072523 – purine–containing compound catabolic process
GO:0007270 – neuron–neuron synaptic transmission
GO:0061138 – morphogenesis of a branching epithelium
REAC:381426 – Regulation of Insulin–like Growth Factor (IGF) transport and uptake by Insulin–like Growth Factor Binding Proteins (IGFBPs)
REAC:4641263 – Regulation of FZD by ubiquitination
GO:0072583 – clathrin–dependent endocytosis
GO:0061213 – positive regulation of mesonephros development
GO:0050768 – negative regulation of neurogenesis
GO:0072006 – nephron development
GO:0008237 – metallopeptidase activity
REAC:881907 – Gastrin–CREB signalling pathway via PKC and MAPK
GO:0001656 – metanephros development
GO:0030552 – cAMP binding
GO:0008081 – phosphoric diester hydrolase activity
GO:0060688 – regulation of morphogenesis of a branching structure
GO:0045665 – negative regulation of neuron differentiation
GO:0090183 – regulation of kidney development
GO:0045600 – positive regulation of fat cell differentiation
GO:0048704 – embryonic skeletal system morphogenesis
GO:0048167 – regulation of synaptic plasticity
GO:0072080 – nephron tubule development
REAC:3000157 – Laminin interactions
GO:0060993 – kidney morphogenesis
GO:0060076 – excitatory synapse
GO:0001763 – morphogenesis of a branching structure
GO:0061326 – renal tubule development
REAC:418555 – G alpha (s) signalling events
REAC:416476 – G alpha (q) signalling events
GO:0009612 – response to mechanical stimulus
REAC:3000178 – ECM proteoglycans
GO:0019722 – calcium–mediated signaling
REAC:392154 – Nitric oxide stimulates guanylate cyclase
GO:0060147 – regulation of posttranscriptional gene silencing
GO:0060966 – regulation of gene silencing by RNA
GO:0001657 – ureteric bud development
GO:0010769 – regulation of cell morphogenesis involved in differentiation
GO:0051961 – negative regulation of nervous system development
GO:0072164 – mesonephric tubule development
GO:0072163 – mesonephric epithelium development
GO:0016339 – calcium–dependent cell–cell adhesion via plasma membrane cell adhesion molecules
GO:0014909 – smooth muscle cell migration
GO:0048754 – branching morphogenesis of an epithelial tube
KEGG:04972 – Pancreatic secretion
GO:0036465 – synaptic vesicle recycling
GO:0048660 – regulation of smooth muscle cell proliferation
GO:0001823 – mesonephros development
KEGG:04072 – Phospholipase D signaling pathway
GO:0009166 – nucleotide catabolic process
GO:0048705 – skeletal system morphogenesis
GO:0010721 – negative regulation of cell development
GO:0005178 – integrin binding
REAC:112316 – Neuronal System
GO:0048608 – reproductive structure development
GO:0050673 – epithelial cell proliferation
GO:1905332 – positive regulation of morphogenesis of an epithelium
GO:0072009 – nephron epithelium development
GO:0001666 – response to hypoxia
GO:0061458 – reproductive system development
GO:0017147 – Wnt–protein binding
GO:0006024 – glycosaminoglycan biosynthetic process
GO:0048661 – positive regulation of smooth muscle cell proliferation
GO:0006023 – aminoglycan biosynthetic process
0
2
4
P-Value

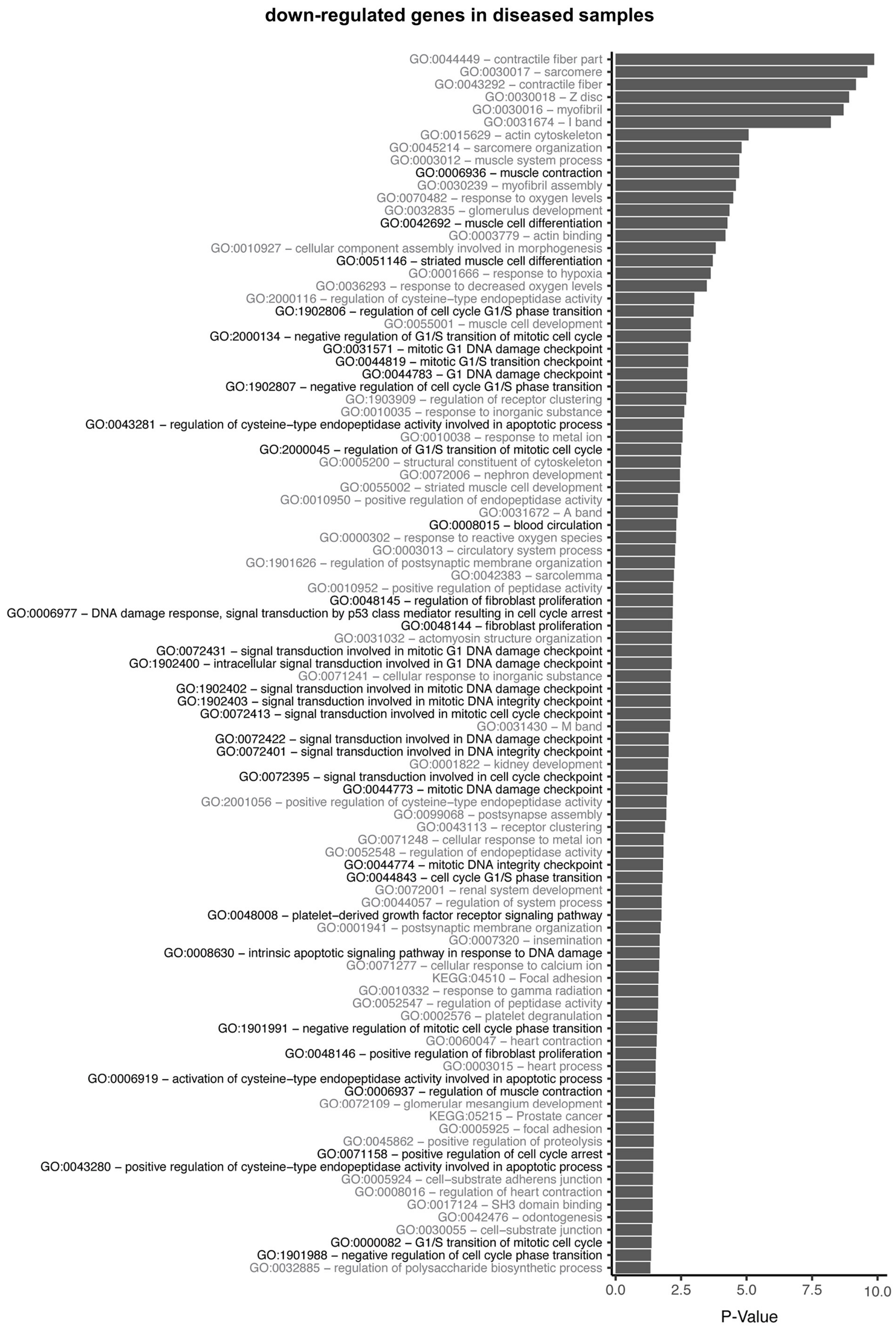
down-regulated genes in diseased samples
GO:0044449 – contractile fiber part
GO:0030017 – sarcomere
GO:0043292 – contractile fiber
GO:0030018 – Z disc
GO:0030016 – myofibril
GO:0031674 – I band
GO:0015629 – actin cytoskeleton
GO:0045214 – sarcomere organization
GO:0003012 – muscle system process
GO:0006936 – muscle contraction
GO:0030239 – myofibril assembly
GO:0070482 – response to oxygen levels
GO:0032835 – glomerulus development
GO:0042692 – muscle cell differentiation
GO:0003779 – actin binding
GO:0010927 – cellular component assembly involved in morphogenesis
GO:0051146 – striated muscle cell differentiation
GO:0001666 – response to hypoxia
GO:0036293 – response to decreased oxygen levels
GO:2000116 – regulation of cysteine−type endopeptidase activity
GO:1902806 – regulation of cell cycle G1/S phase transition
GO:0055001 – muscle cell development
GO:2000134 – negative regulation of G1/S transition of mitotic cell cycle
GO:0031571 – mitotic G1 DNA damage checkpoint
GO:0044819 – mitotic G1/S transition checkpoint
GO:0044783 – G1 DNA damage checkpoint
GO:1902807 – negative regulation of cell cycle G1/S phase transition
GO:1903909 – regulation of receptor clustering
GO:0010035 – response to inorganic substance
GO:0043281 – regulation of cysteine−type endopeptidase activity involved in apoptotic process
GO:0010038 – response to metal ion
GO:2000045 – regulation of G1/S transition of mitotic cell cycle
GO:0005200 – structural constituent of cytoskeleton
GO:0072006 – nephron development
GO:0055002 – striated muscle cell development
GO:0010950 – positive regulation of endopeptidase activity
GO:0031672 – A band
GO:0008015 – blood circulation
GO:0000302 – response to reactive oxygen species
GO:0003013 – circulatory system process
GO:1901626 – regulation of postsynaptic membrane organization
GO:0042383 – sarcolemma
GO:0010952 – positive regulation of peptidase activity
GO:0048145 – regulation of fibroblast proliferation
GO:0006977 – DNA damage response, signal transduction by p53 class mediator resulting in cell cycle arrest
GO:0048144 – fibroblast proliferation
GO:0031032 – actomyosin structure organization
GO:0072431 – signal transduction involved in mitotic G1 DNA damage checkpoint
GO:1902400 – intracellular signal transduction involved in G1 DNA damage checkpoint
GO:0071241 – cellular response to inorganic substance
GO:1902402 – signal transduction involved in mitotic DNA damage checkpoint
GO:1902403 – signal transduction involved in mitotic DNA integrity checkpoint
GO:0072413 – signal transduction involved in mitotic cell cycle checkpoint
GO:0031430 – M band
GO:0072422 – signal transduction involved in DNA damage checkpoint
GO:0072401 – signal transduction involved in DNA integrity checkpoint
GO:0001822 – kidney development
GO:0072395 – signal transduction involved in cell cycle checkpoint
GO:0044773 – mitotic DNA damage checkpoint
GO:2001056 – positive regulation of cysteine−type endopeptidase activity
GO:0099068 – postsynapse assembly
GO:0043113 – receptor clustering
GO:0071248 – cellular response to metal ion
GO:0052548 – regulation of endopeptidase activity
GO:0044774 – mitotic DNA integrity checkpoint
GO:0044843 – cell cycle G1/S phase transition
GO:0072001 – renal system development
GO:0044057 – regulation of system process
GO:0048008 – platelet−derived growth factor receptor signaling pathway
GO:0001941 – postsynaptic membrane organization
GO:0007320 – insemination
GO:0008630 – intrinsic apoptotic signaling pathway in response to DNA damage
GO:0071277 – cellular response to calcium ion
KEGG:04510 – Focal adhesion
GO:0010332 – response to gamma radiation
GO:0052547 – regulation of peptidase activity
GO:0002576 – platelet degranulation
GO:1901991 – negative regulation of mitotic cell cycle phase transition
GO:0060047 – heart contraction
GO:0048146 – positive regulation of fibroblast proliferation
GO:0003015 – heart process
GO:0006919 – activation of cysteine−type endopeptidase activity involved in apoptotic process
GO:0006937 – regulation of muscle contraction
GO:0072109 – glomerular mesangium development
KEGG:05215 – Prostate cancer
GO:0005925 – focal adhesion
GO:0045862 – positive regulation of proteolysis
GO:0071158 – positive regulation of cell cycle arrest
GO:0043280 – positive regulation of cysteine−type endopeptidase activity involved in apoptotic process
GO:0005924 – cell−substrate adherens junction
GO:0008016 – regulation of heart contraction
GO:0017124 – SH3 domain binding
GO:0042476 – odontogenesis
GO:0030055 – cell−substrate junction
GO:0000082 – G1/S transition of mitotic cell cycle
GO:1901988 – negative regulation of cell cycle phase transition
GO:0032885 – regulation of polysaccharide biosynthetic process
0.0
2.5
5.0
7.5
10.0
P-Value

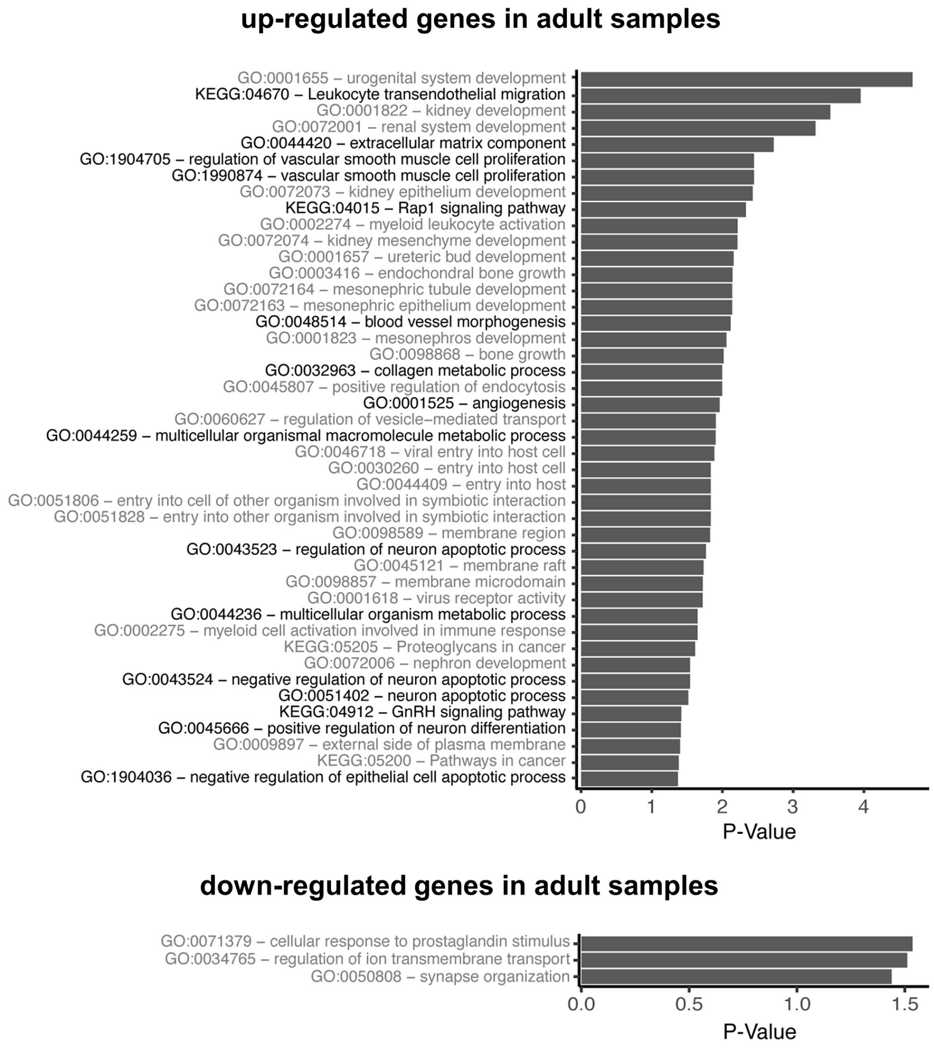

Figure S6. Gene Ontology (GO) terms of biological processes enriched in hTERT transduced PDGFRα+cMSCs. GO analysis of differentially expressed genes by RNA-sequencing (RNA-seq) in hTERT transduced PDGFRα+cMSCs compared to non-transduced PDGFRα+cMSCs and empty vector controls. The significantly ($p<0.05$) up-regulated and down-regulated biological processes and molecular functions categories are shown. Full RNA-seq data can be accessed at gene expression omnibus; accession number GSE112297. See also Figure 4.

Figure S7.

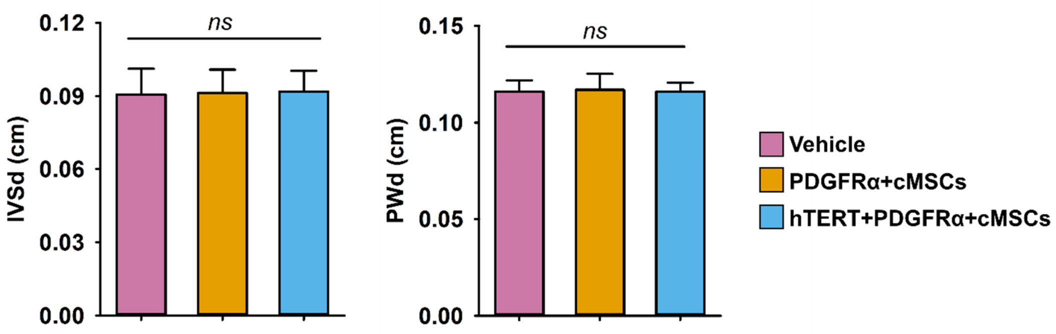

Figure S7. Measurements of intraventricular septal wall thickness in diastole (IVSd) and posterior wall thickness in diastole (PWd) in infarcted hearts 1-month after transplantation. There are no significant differences in IVSd (*left*) and PWd (*right*) in non-hTERT (PDGFRα+cMSCs) and hTERT-transduced cells (hTERT+PDGFRα+cMSCs) treated animals compared to vehicle controls.

Figure S8.

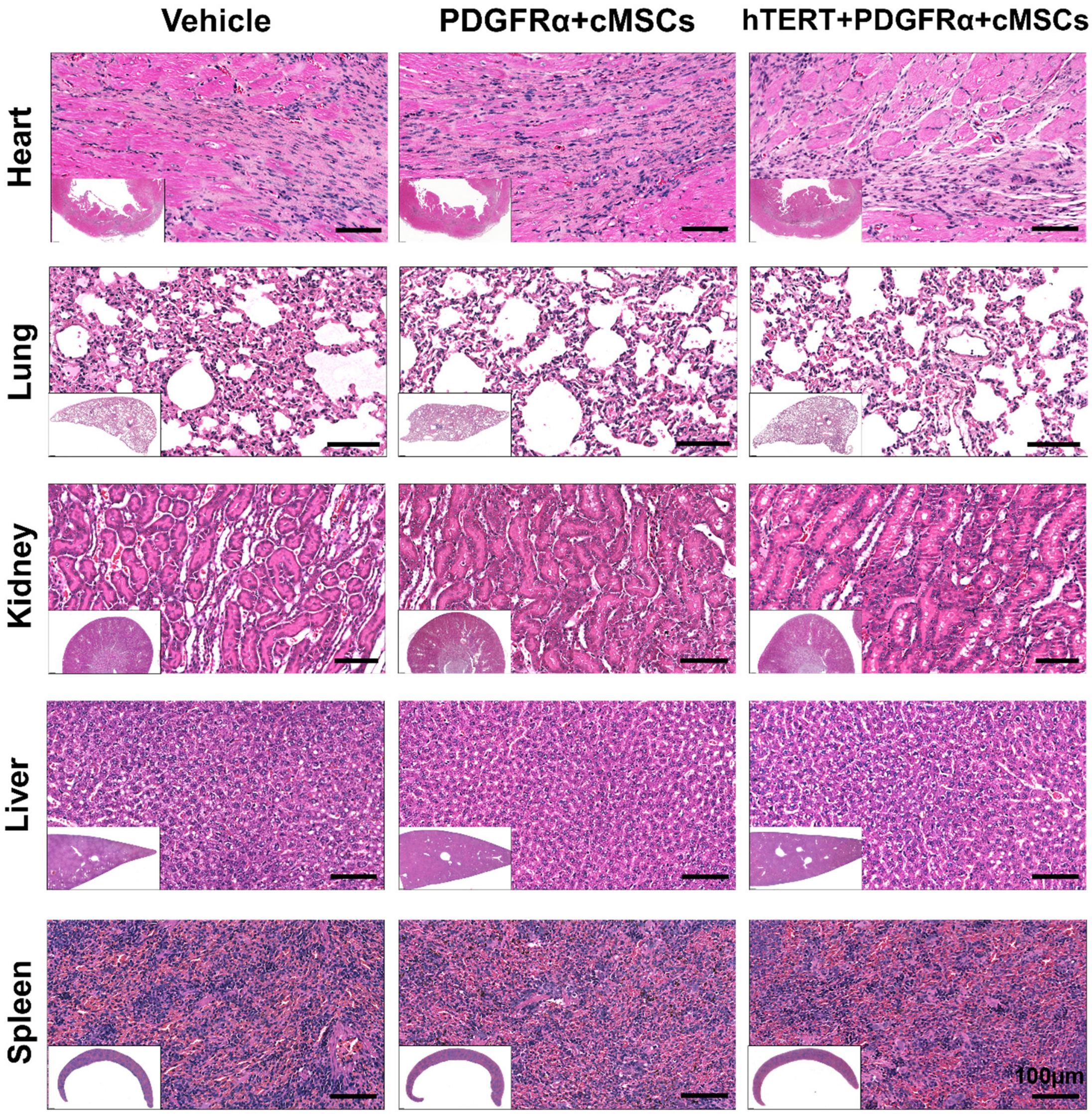

Figure S8. hTERT+PDGFRα+cMSC transplantation does not cause tumorgenicity. Representative images of hematoxylin and eosin (H&E) staining of heart, lung, spleen, kidney and liver cross sections are shown. Representative H&E images demonstrate that both PDGFRα+cMSC and hTERT+PDGFRα+cMSC treatment did not change the normal structure or produce any tumours.

Figure S9.

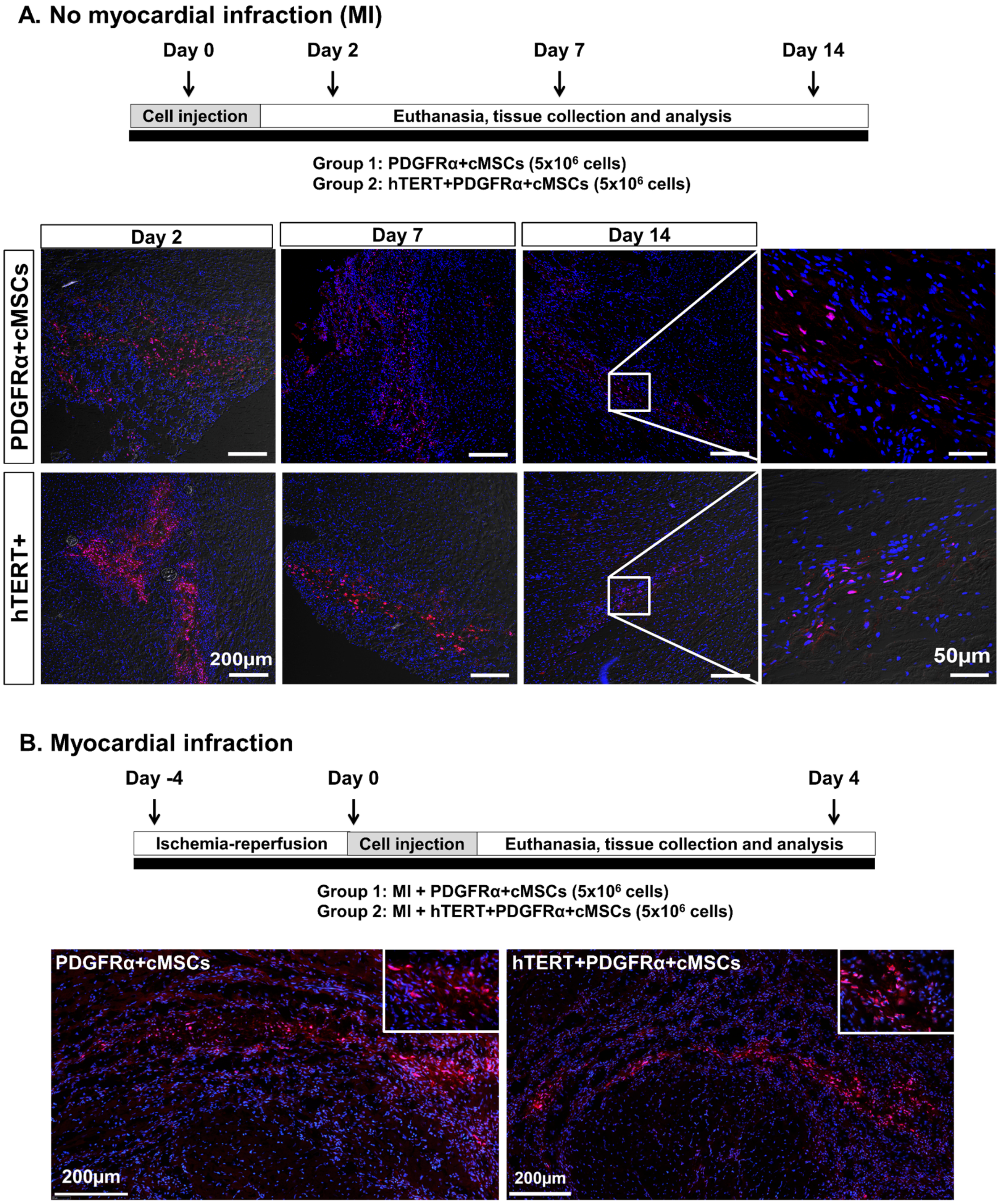

Figure S9. Engraftment capacity of non-hTERT and hTERT transduced PDGFRα+cMSCs in non-injured (no myocardial infarction [no MI]) and infarcted (MI) hearts. (A) Human PDGFRα+cMSCs and hTERT+PDGFRα+cMSCs were injected into non-injured athymic rat hearts (no MI). Immunostaining against human nuclei was used to identify human cells. Representative confocal immunofluorescence images demonstrating engrafted cells at 2, 7 and 14 days after transplantation. A higher number of human cells was seen at 2 days and minimal human cells were seen at 14 days. The area enclosed by the white box is magnified in the right panel: Pink = human nuclei (colocalised with DAPI), Blue = DAPI-stained nuclei. **(B)** Representative confocal immunofluorescence images demonstrating engrafted PDGFRα+cMSCs and hTERT+PDGFRα+cMSCs into infarcted area at 4 days after transplantation. See also Figure 5J for engraftment ratio.

Figure S10.

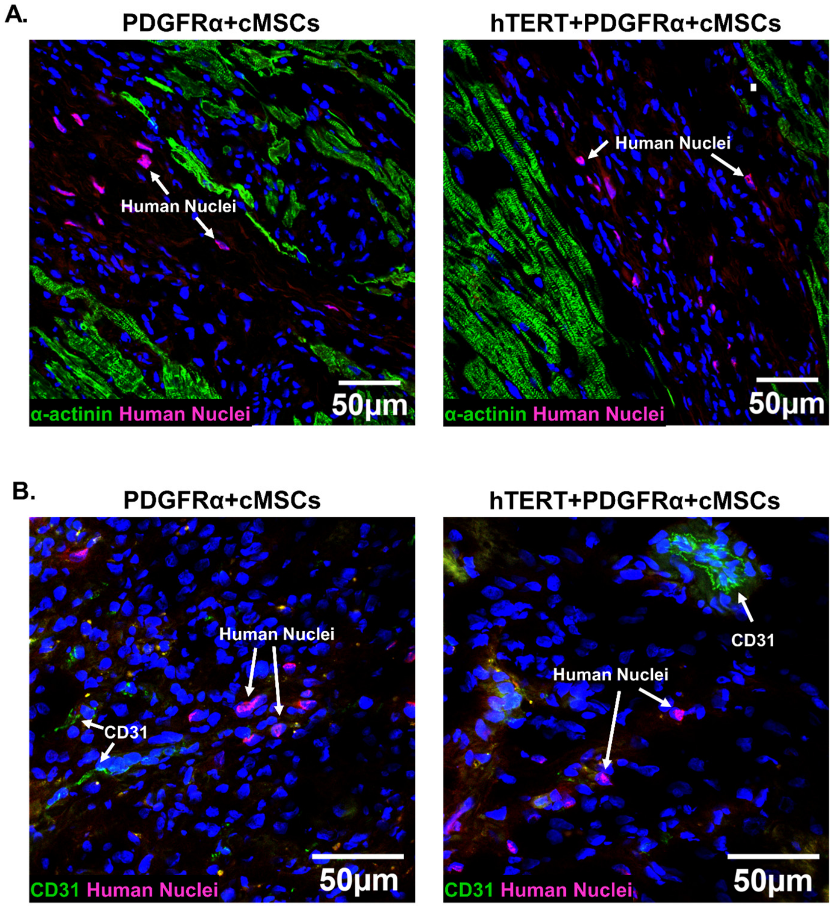

Figure S10. Transplanted PDGFRα+cMSCs are not capable of *in vivo* differentiation to endothelial and cardiomyocyte lineages. **(A)** Representative confocal immunofluorescence images of co-immunostaining against human nuclei (pink, colocalised with DAPI) and the cardiomyocyte marker α-actinin (green) in the rat myocardium two weeks after transplantation. No evidence of non-transduced or hTERT over-expressing PDGFRα+cMSC differentiation into the cardiomyocyte lineage. DAPI-stained nuclei in blue. **(B)** Representative confocal immunofluorescence images of co-immunostaining against human nuclei (pink, colocalised with DAPI) and the endothelial cell marker CD31 (green) in the rat myocardium. Nuclei stained in blue with DAPI. No evidence of human cells differentiation into the endothelial lineage.

Figure S11.

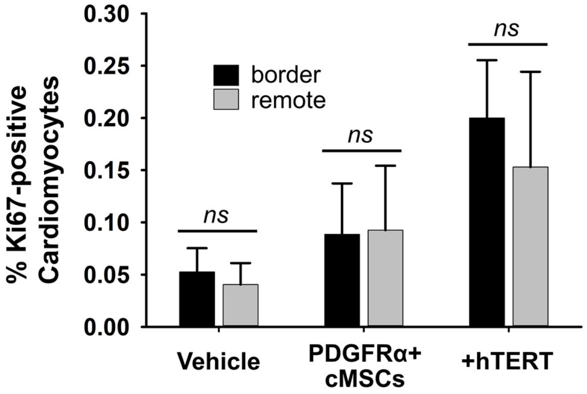

Figure S11. Quantification of the percentage of Ki67-positive cardiomyocytes (CMs) between border and remote areas are shown. Although there are increased Ki67-positive CMs in rats that received hTERT+PDGFRα+cMSCs, no significant difference in the Ki67-positive CMs between border areas and remote areas were observed in all treatment groups.

Figure S12.

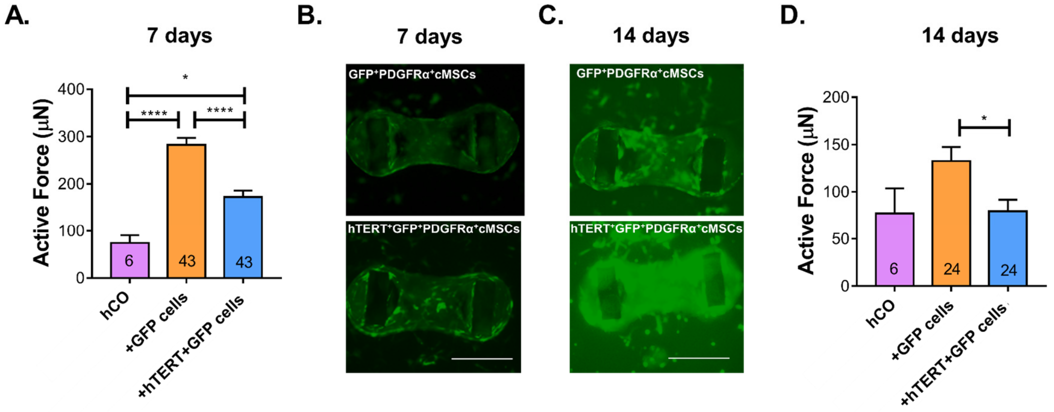

Figure S12. hTERT+PDGFRα+cMSCs enhances human cardiomyocyte force of contraction using organ bath. Non-hTERT and hTERT-transduced PDGFRα+cMSCs were co-cultured with human pluripotent stem cell-derived cardiac organoids (hCOs). **(A)** GFP-transduced PDGFRα+cMSCs and hTERT over-expressing PDGFRα+cMSCs increase the hCO force of contraction compared to controls. Data presented as Mean ± SE; N = 6-43 pooled from 2 different patient samples. *P < 0.05, ****P <0.0001, using one-way ANOVA with Tukey's post-test. **(B)** GFP images of hCO at day 7 indicating that hTERT overexpressing GFP-transduced PDGFRα+cMSCs are more populous than GFP-transduced PDGFRα+cMSCs. **(C)** GFP images of hCO at day 14 indicating that hTERT overexpressing GFP-transduced PDGFRα+cMSCs and GFP-transduced PDGFRα+cMSCs proliferate through the hCO. **(D)** Functional benefits of GFP-transduced PDGFRα+cMSCs and hTERT over-expressing PDGFRα+cMSCs are lost by day 14 due to too much proliferation (especially in the hTERT). Data presented as Mean ± SE; N = 6-24 pooled from 2 different patient samples. *P < 0.05, using one-way ANOVA with Tukey's post-test.

Supplementary References

1 Pickett, H. A., Cesare, A. J., Johnstone, R. L., Neumann, A. A. & Reddel, R. R. Control of telomere length by a trimming mechanism that involves generation of t-circles. *EMBO J.* **28**, 799-809 (2009).

2 Lee, M. *et al.* Telomere extension by telomerase and ALT generates variant repeats by mechanistically distinct processes. *Nucleic Acids Res* **42**, 1733-1746, doi:10.1093/nar/gkt1117 (2014).

3 Le, T. Y. L. *et al.* Platelet-Derived Growth Factor Receptor-Alpha Expressing Cardiac Progenitor Cells Can Be Derived from Previously Cryopreserved Human Heart Samples. *Stem Cells Dev* **27**, 184-198, doi:10.1089/scd.2017.0082 (2018).

4 Ashton, A. W. *et al.* Role of Nongenomic Signaling Pathways Activated by Aldosterone During Cardiac Reperfusion Injury. *Mol Endocrinol* **29**, 1144-1155, doi:10.1210/ME.2014-1410 (2015).

5 Chong, J. J. *et al.* Adult cardiac-resident MSC-like stem cells with a proepicardial origin. *Cell Stem Cell* **9**, 527-540, doi:10.1016/j.stem.2011.10.002 (2011).

6 Chong, J. J. *et al.* Progenitor cells identified by PDGFR-alpha expression in the developing and diseased human heart. *Stem Cells Dev* **22**, 1932-1943, doi:10.1089/scd.2012.0542 (2013).

7 Mills, R. J. *et al.* Functional screening in human cardiac organoids reveals a metabolic mechanism for cardiomyocyte cell cycle arrest. *Proc Natl Acad Sci U S A* **114**, E8372-E8381, doi:10.1073/pnas.1707316114 (2017).

8 Voges, H. K. *et al.* Development of a human cardiac organoid injury model reveals innate regenerative potential. *Development* **144**, 1118-1127, doi:10.1242/dev.143966 (2017).

9 Mills, R. J., Voges, H. K., Porrello, E. R. & Hudson, J. E. Cryoinjury Model for Tissue Injury and Repair in Bioengineered Human Striated Muscle. *Methods Mol Biol* **1668**, 209-224, doi:10.1007/978-1-4939-7283-8_15 (2017).

10 Hudson, J., Titmarsh, D., Hidalgo, A., Wolvetang, E. & Cooper-White, J. Primitive cardiac cells from human embryonic stem cells. *Stem Cells Dev* **21**, 1513-1523, doi:10.1089/scd.2011.0254 (2012).

11 Hudson, J. E., Brooke, G., Blair, C., Wolvetang, E. & Cooper-White, J. J. Development of myocardial constructs using modulus-matched acrylated polypropylene glycol triol substrate and different nonmyocyte cell populations. *Tissue Eng Part A* **17**, 2279-2289, doi:10.1089/ten.TEA.2010.0743 (2011).

12 Tiburcy, M. *et al.* Defined Engineered Human Myocardium With Advanced Maturation for Applications in Heart Failure Modeling and Repair. *Circulation* **135**, 1832-1847, doi:10.1161/CIRCULATIONAHA.116.024145 (2017).
